# Dye-Sensitized Solar Cells: Fundamentals and Current Status

**DOI:** 10.1186/s11671-018-2760-6

**Published:** 2018-11-28

**Authors:** Khushboo Sharma, Vinay Sharma, S. S. Sharma

**Affiliations:** 1 Department of Physics, Bhagwant University, Ajmer, 305004 India; 2 School of Materials Science and Engineering, Nanyang Technological University, Singapore, 639798 Singapore; 3 Department of Physics, Govt. Women Engineering College, Ajmer, 305002 India

**Keywords:** Dye-sensitized solar cells (DSSCs), Photoanode, Counter electrode, Electrolytes, Metal and metal-free organic dyes, Efficiency, Stability

## Abstract

Dye-sensitized solar cells (DSSCs) belong to the group of thin-film solar cells which have been under extensive research for more than two decades due to their low cost, simple preparation methodology, low toxicity and ease of production. Still, there is lot of scope for the replacement of current DSSC materials due to their high cost, less abundance, and long-term stability. The efficiency of existing DSSCs reaches up to 12%, using Ru(II) dyes by optimizing material and structural properties which is still less than the efficiency offered by first- and second-generation solar cells, i.e., other thin-film solar cells and Si-based solar cells which offer ~ 20–30% efficiency. This article provides an in-depth review on DSSC construction, operating principle, key problems (low efficiency, low scalability, and low stability), prospective efficient materials, and finally a brief insight to commercialization.

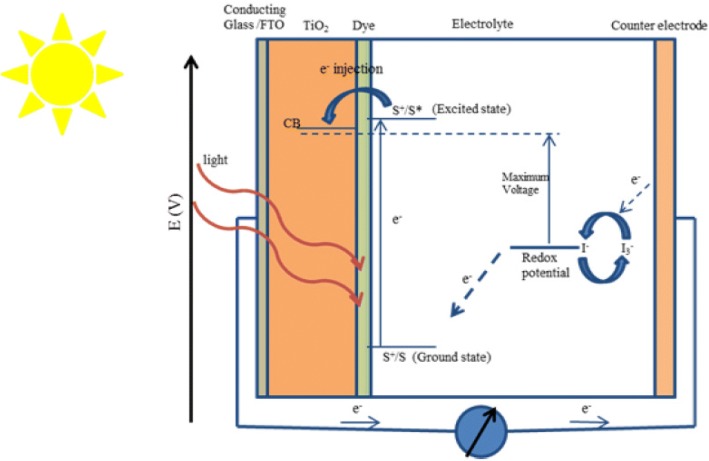

## Introduction

Dye-sensitized solar cells (DSSCs) have arisen as a technically and economically credible alternative to the p-n junction photovoltaic devices. In the late 1960s, it was discovered that electricity can be generated through illuminated organic dyes in electrochemical cells. At the University of California at Berkeley, chlorophyll was extracted from spinach (photosynthesis). First chlorophyll-sensitized zinc oxide (ZnO) electrode was synthesized in 1972. For the first time, through electron injection of excited dye molecules into a wide band gap of semiconductor, photons were converted into electricity [1]. A lot of research has been done on ZnO-single crystals [2], but the efficiency of these dye-sensitized solar cells was very poor, as the monolayer of dye molecules was able to absorb incident light only up to 1%. Thus, the efficiency was improved by optimizing the porosity of the electrode made up of fine oxide powder, so that the absorption of dye over electrode could be enhanced and as a result light harvesting efficiency (LHE) could also be enhanced. As a result, nanoporous titanium dioxide (TiO_2_) electrodes with a roughness factor of ca.1000 were discovered, and in 1991, DSSCs with 7% efficiency were invented [3]. These cells, also known as Grätzel cells, were originally co-invented in 1988 by Brian O’Regan and Michael Grätzel at UC Berkeley [3] and were further developed by the aforementioned scientists at Ecole Polytechnique Fédèrale de Lausanne (EPFL) till 1991.

Brian O’Regan and Michael Grätzel fabricated a device based on a 10-μm-thick, high surface area and optically transparent film of TiO_2_ nanoparticles, coated with a monolayer of a charge transfer dye with ideal spectral characteristics to sensitize the film for light harvesting. The device harvested a high proportion of the incident solar energy flux of 46% and showed exceptionally high efficiencies, even more than 80% efficiencies for the conversion of incident photons to electrical current. The overall incident photon to current conversion efficiency (IPCE) yield was 7.1–7.9% in simulated solar light and 12% in diffuse daylight. A large short circuit current density *J*_SC_ (greater than 12 mAcm^− 2^) and exceptional stability (sustaining at least five million turnovers without decomposition) and low cost made the practical application feasible [3]. In 1993, Grätzel et al. reported 9.6% efficiency of cells, and then in 1997, they achieved 10% at the National Renewable Energy Laboratory (NREL). The sensitizers are usually designed to have functional groups such as –COOH, –PO_3_H_2_, and –B(OH)_2_ for stable adsorption onto the semiconductor substrate [4, 5]. Recently in 2018, an efficiency of 8.75% was reported for hybrid dye-titania nanoparticle-based DSSC for superior low temperature by Costa et al. [6]. In a traditional solar cell, Si provides two functions: acts as source of photoelectrons and provides electric field to separate the charges and create a current. But, in DSSCs, the bulk of semiconductor is only used as a charge transporter and the photoelectrons are provided by photosensitive dyes. The theoretically predicted power conversion efficiency (PCE) of DSSCs was approximately 20% [7, 8]; thus, an extensive research has been made over the years on DSSCs to improve the efficiency and to augment its commercialization. However, in the last few decades, a lot of experiments were carried out to improve the performance of DSSCs. For instance, if one goes through the review articles or papers published around 1920 and 1921, a remarkable difference may be observed in the performance as well as fabrication of these cells. Few review papers are discussed below with the objective and main results shown in a respective article to get an idea how the performance of these cells has been improved and, thus, how the DSSCs became a hot topic for researchers.

Anandan reviewed the improvements and arising challenges in dye-sensitized solar cells till 2007 [9]. The main components of his review study were light harvesting inorganic dye molecules, p-CuO nanorod counter electrodes, and self-organization of electroactive polymers, and he showed how these materials perform in a rationally designed solar cell. However, the maximum IPCE of 7% was discussed in the review paper for naphthyridine coordinated Ru complex [10] which was good till 2007 but is almost half to the efficiencies shown in later work.

**Fig. 1:**
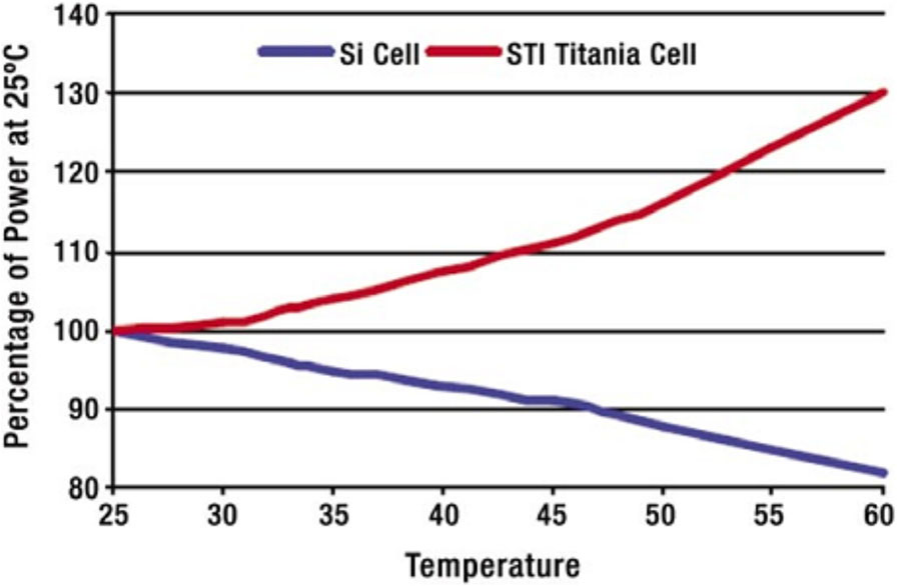
The performance of dye PV modules increases with temperature, contrary to Si-based modules [(Web reference [available online at http://www.sta.com.au/downloads/DSC%20Booklet.pdf] [11, 12]

The main emphasis of the review paper published by Bose et al. [11] was the current state and developments in the field of photoelectrode, photosensitizer, and electrolyte for DSSCs till 2015. They have included an interesting study of comparing the performance of the DSSC module with that of the Si-based module by the graph shown in Fig. 1 [12] and concluded that the performance of the DSSC module is far better than that of the Si module. Also, the highest efficiency discussed in this review paper was 11.2% for N719 dye-based DSSC.

Shalini et al. [13] emphasized on sensitizers, including ruthenium complexes, metal-free organic dyes, quantum-dot sensitizer, perovskite-based sensitizer, mordant dyes, and natural dyes. However, this article provides a great knowledge about the different types of sensitizers, but lacks the information regarding other important components of the DSSCs. Again, apart from discussing all different components of DSSCs, the review article by Jihuai Wu et al. [14] was concentrated over the counter electrode part. They have discussed the study of different types of counter electrodes based on transparency and flexibility, metals and alloys, carbon materials, conductive polymers, transition metal compounds, and hybrids. A highest efficiency of 14.3% was discussed for the DSSC fabricated with Au/GNP as a counter electrode, Co^3+/2+^ as a redox couple, and LEG4 + ADEKA-1 as a sensitizer [15] and was shown in the review article. Similarly, Yeoh et al. and Fan et al. [16, 17] have given a brief review over the photoanode of DSSC. They have classified modification of photoanode into three categories, namely interfacial modification through the introduction of blocking and scattering layer, compositing, doping with non-metallic anions and metallic cations, interfacial engineering, and replacing the conventional mesoporous semiconducting metal oxide films like with 1-D or 2-D nanostructures.

Thus, by comparing different review articles published earlier, it can be easily seen that the present review article “Dye Sensitized solar Cells: Fundamentals and Current Status” gives the in-depth study of different components and their application in DSSCs as well as construction and working of these cells.

## Construction and Working of DSSCs

**Fig. 2:**
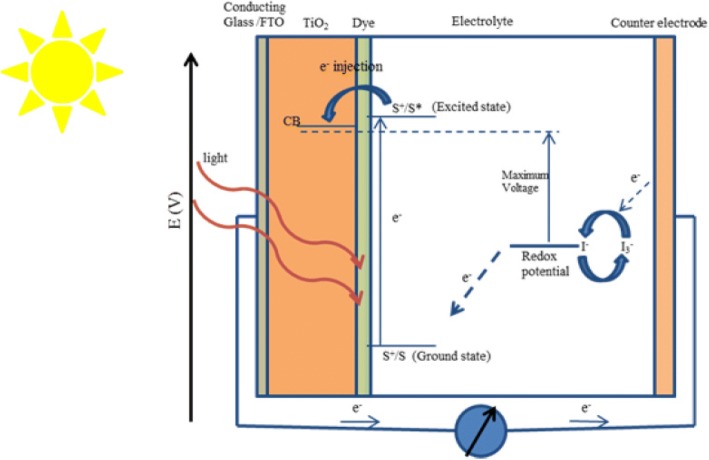
Construction and working principle of the dye-sensitized nanocrystalline solar cells

The working electrode, sensitizer (dye), redox-mediator (electrolyte), and counter electrode are four key parameters for a DSSC. DSSC is an assembly of working electrode soaked with a sensitizer or a dye and sealed to the counter electrode soaked with a thin layer of electrolyte with the help of a hot melt tape to prevent the leakage of the electrolyte (as shown in Fig. 2). The components as well as the construction and working of DSSCs are shown below:

### Transparent and Conductive Substrate

DSSCs are typically constructed with two sheets of conductive transparent materials, which help a substrate for the deposition of the semiconductor and catalyst, acting also as current collectors [18, 19] There are two main characteristics of a substrate being used in a DSSC: Firstly, more than 80% of transparency is required by the substrate to permit the passage of optimum sunlight to the effective area of the cell. Secondly, for the efficient charge transfer and reduced energy loss in DSSCs, it should have a high electrical conductivity. The fluorine-doped tin oxide (FTO, SnO_2_: F) and indium-doped tin oxide (ITO, In_2_O_3_: Sn) are usually applied as a conductive substrate in DSSCs. These substrates consist of soda lime glass coated with the layers of indium-doped tin oxide and fluorine-doped tin oxide. The ITO films have a transmittance > 80% and 18 Ω/cm^2^ of sheet resistance, while FTO films show a lower transmittance of ~ 75% in the visible region and sheet resistance of 8.5 Ω/cm^2^ [18].

### Working Electrode (WE)

The working electrodes (WE) are prepared by depositing a thin layer of oxide semiconducting materials such as TiO_2_, Nb_2_O_5_, ZnO, SnO_2_ (n-type), and NiO (p-type) on a transparent conducting glass plate made of FTO or ITO. These oxides have a wide energy band gap of 3–3.2 eV. The application of an anatase allotropic form of TiO_2_ is more commendable in DSSCs as compared to a rutile form due to its higher energy band gap of 3.2 eV whereas the rutile form has a band gap of about 3 eV [20, 21], although alternative wide band gap oxides such as ZnO and Nb_2_O_5_ have also given promising results [22, 23]. Due to being non-toxic and less expensive and its easy availability, TiO_2_ is mostly used as a semiconducting layer. However, these semiconducting layers absorb only a small fraction of light in the UV region; hence, these working electrodes are then immersed in a mixture of a photosensitive molecular sensitizer and a solvent. After soaking the film within the dye solution, the dye gets covalently bonded to the TiO_2_ surface. Due to the highly porous structure and the large surface area of the electrode, a high number of dye molecules get attached on the nanocrystalline TiO_2_ surface, and thus, light absorption at the semiconductor surface increases.

### Photosensitizer or Dye

Dye is the component of DSSC responsible for the maximum absorption of the incident light. Any material being dye should have the following photophysical and electrochemical properties:

1. Firstly, the dye should be luminescent.
2. Secondly, the absorption spectra of the dye should cover ultraviolet-visible (UV-vis) and near-infrared region (NIR) regions.
3. The highest occupied molecular orbital (HOMO) should be located far from the surface of the conduction band of TiO_2_ and the lowest unoccupied molecular orbital (LUMO) should be placed as close to the surface of the TiO_2_, and subsequently should be higher with respect to the TiO_2_ conduction band potential.
4. HOMO should lie lower than that of redox electrolytes.
5. The periphery of the dye should be hydrophobic to enhance the long-term stability of cells, as it results in minimized direct contact between electrolyte and anode; otherwise, water-induced distortion of the dye from the TiO_2_ surface can appear which may reduce the stability of cells.
6. To avoid the aggregation of the dye over the TiO_2_ surface, co-absorbents like chenodeoxycholic acid (CDCA) and anchoring groups like alkoxy-silyl [24], phosphoric acid [25], and carboxylic acid group [26, 27] were inserted between the dye and TiO_2_. This results in the prevention of dye aggregation and thus limits the recombination reaction [28] between redox electrolyte and electrons in the TiO_2_ nanolayer as well as results in the formation of stable linkage.

### Electrolyte

An electrolyte (such as I^−^/I^−^_3_, Br^−^/Br^−^ _2_ [29], SCN^−^/SCN_2_ [30], and Co(II)/Co(III) [31]) has five main components, i.e., redox couple, solvent, additives, ionic liquids, and cations. The following properties should be present in an electrolyte:

1. Redox couple should be able to regenerate the oxidized dye efficiently.
2. Should have long-term chemical, thermal, and electrochemical stability.
3. Should be non-corrosive with DSSC components.
4. Should be able to permit fast diffusion of charge carriers, enhance conductivity, and create effective contact between the working and counter electrodes.
5. Absorption spectra of an electrolyte should not overlap with the absorption spectra of a dye.

I^−^/I^−^ _3_ has been demonstrated as a highly efficient electrolyte [32], but there are certain limitations associated with its application in DSSCs. I^−^/I^−^ _3_ electrolyte corrodes glass/TiO_2_/Pt; it is highly volatile and responsible for photodegradation and dye desorption and has poor long-term stability [33, 34]. Acetonitrile (ACN), *N*-methylpyrrolidine (NMP), and solvent mixtures, such as ACN/valeronitrile, have been used as a solvent having high dielectric constants. 4-Tert-butylpyridine (TBP) is mostly used as an additive to shift the conduction band of TiO_2_ upwards, which results in an increase in the value of open circuit voltage (*V*_OC_), reduced cell photocurrent (*J*_SC_), and less injection driving force. It is believed that TBP on a TiO_2_ surface reduces recombination through back transfer to an electrolyte [35]. However, the biggest drawback allied with the ionic liquid is their leakage factor. Thus, solid-state electrolytes are developed to avoid the drawbacks associated with ionic liquid (IL) electrolytes [36]. Also, to test the failure of the redox electrolyte or the sealing under long-term illumination, long-term light soaking tests on sealed cells have also progressed significantly over the years [37].

### Counter Electrode (CE)

CE in DSSCs are mostly prepared by using platinum (Pt) or carbon (C). Both working and counter electrodes are sealed together, and subsequently, an electrolyte is filled with a help of a syringe. Counter electrode catalyzes the reduction of I^−^/I^−^ _3_ liquid electrolyte and collects holes from the hole transport materials (HTMs). Pt is used mostly as a counter electrode as it demonstrates higher efficiencies [38], but the replacement of Pt was much needed due to its higher cost and less abundance. Thus, several alternatives have developed to replace Pt in DSSCs, such as carbon [39], carbonylsulfide (CoS) [40], Au/GNP [15], alloy CEs like FeSe [41], and CoNi_0.25_ [42], although the different types of the CEs are also discussed by Jihuai Wu et al. [14].

## Working Principle

The working principle of DSSC involves four basic steps: light absorption, electron injection, transportation of carrier, and collection of current. The following steps are involved in the conversion of photons into current (as shown in Fig. 2):

1. Firstly, the incident light (photon) is absorbed by a photosensitizer, and thus, due to the photon absorption, electrons get promoted from the ground state (S^+^/S) to the excited state (S^+^/S*) of the dye, where the absorption for most of the dye is in the range of 700 nm which corresponds to the photon energy almost about 1.72 eV.
2. Now, the excited electrons with a lifetime of nanosecond range are injected into the conduction band of nanoporous TiO_2_ electrode which lies below the excited state of the dye, where the TiO_2_ absorbs a small fraction of the solar photons from the UV region [43]. As a result, the dye gets oxidized.
  $$ {\mathrm{S}}^{+}/\mathrm{S}+\mathrm{h}\upnu \rightarrow {\mathrm{S}}^{+}/{\mathrm{S}}^{\ast } $$
  $$ {\mathrm{S}}^{+}/{\mathrm{S}}^{\ast}\rightarrow {\mathrm{S}}^{+}/\mathrm{S}+{\mathrm{e}}^{-}\ \left({\mathrm{TiO}}_2\right) $$
3. These injected electrons are transported between TiO_2_ nanoparticles and diffuse towards the back contact (transparent conducting oxide [TCO]). Through the external circuit, electrons reach at the counter electrode.
4. The electrons at the counter electrode reduce I^−^ _3_ to I^−^; thus, dye regeneration or the regeneration of the ground state of the dye takes place due to the acceptance of electrons from I^−^ ion redox mediator, and I^−^ gets oxidized to I^−^ _3_ (oxidized state).
  $$ {\mathrm{S}}^{+}/{\mathrm{S}}^{\ast }+{\mathrm{e}}^{-}\rightarrow {\mathrm{S}}^{+}/\mathrm{S} $$
5. Again, the oxidized mediator (I^−^ _3_) diffuses towards the counter electrode and reduces to I ion.
  $$ {{\mathrm{I}}^{-}}_3+{2\mathrm{e}}^{-}\rightarrow {3\mathrm{I}}^{-} $$

## Evaluation of Dye-Sensitized Solar Cell Performance

**Fig. 3:**
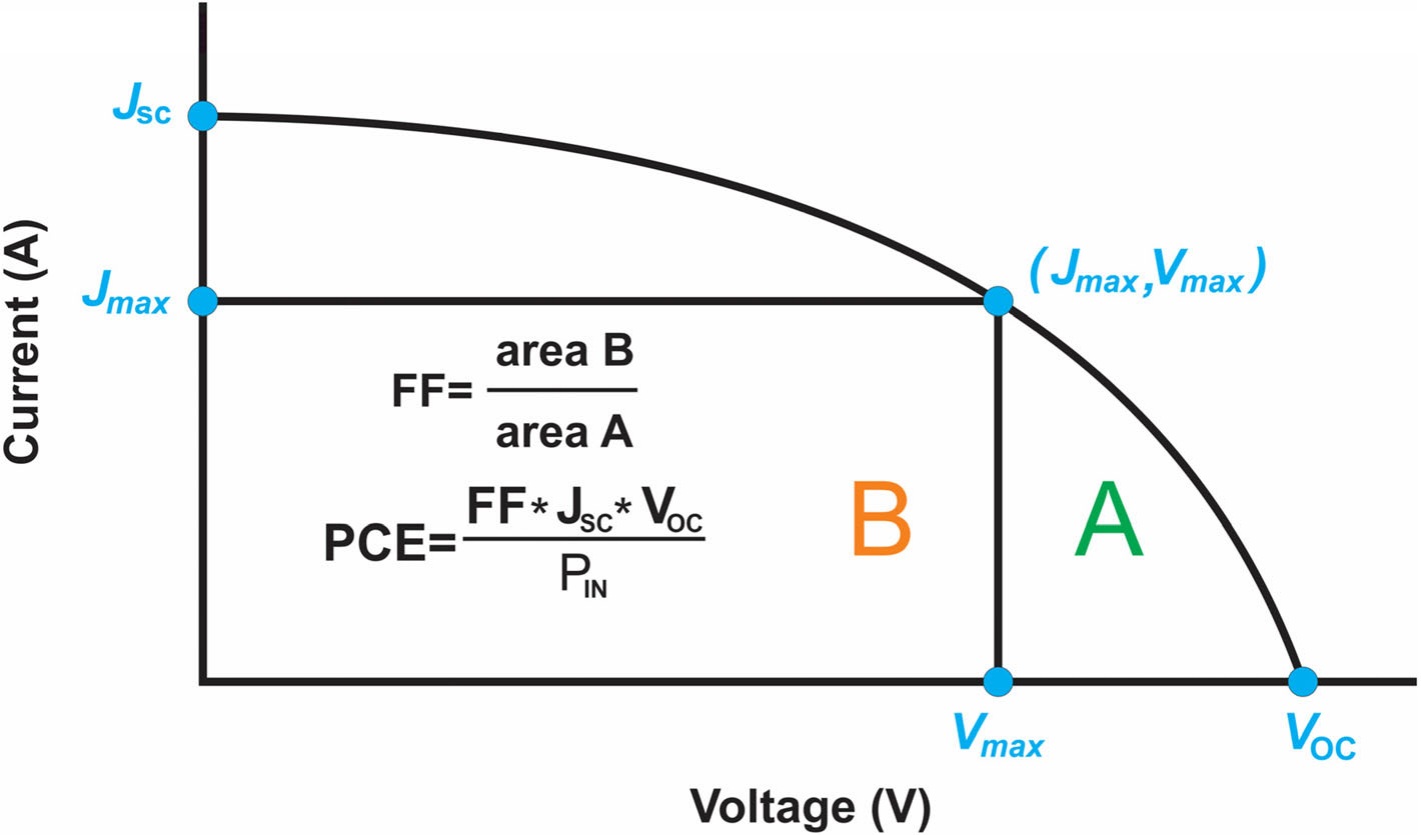
*I–V* curve to evaluate the cells performance

The performance of a dye-sensitized solar cell can be evaluated by using incident photon to current conversion efficiency (IPCE, %), short circuit current (*J*_SC_, mAcm^− 2^), open circuit voltage (*V*_OC_, *V*), maximum power output [*P*_max_], overall efficiency [*η*, %], and fill factor [FF] (as shown in Fig. 3) at a constant light level exposure as shown in Eq. 1 [44].

The current produces when negative and positive electrodes of the cell are short circuited at a zero mV voltage. *V*_OC_ (*V*) is the voltage across negative and positive electrodes under open circuit condition at zero milliampere (mA) current or simply, the potential difference between the conduction band energy of semiconducting material and the redox potential of electrolyte. *P*_max_ is the maximum efficiency of the DSSC to convert sunlight into electricity. The ratio of maximum power output (*J*_mp_ × *V*_mp_) to the product (*V*_OC_ × *J*_SC_) gives FF.

$$ \mathrm{FF}=\frac{\mathrm{Area}\ A}{\mathrm{Area}\ B}=\frac{J_{\mathrm{mp}}\times {V}_{\mathrm{mp}}}{J_{SC}\times {V}_{OC}} $$

Also, the overall efficiency (%) is the percentage of the solar energy (shining on a photovoltaic [PV] device) converting into electrical energy, where *ɳ* increases with the decrease in the value of *J*_SC_ and increase in the values of *V*_OC_, FF, and molar coefficient of dye, respectively.

1 $$ \eta\ \left(\%\right)=\frac{J_{\mathrm{SC}}\times {V}_{\mathrm{OC}}\times \mathrm{FF}}{P_{in}} $$

External quantum efficiency (also known as IPCE) is the ratio of number of electrons flowing through the external circuit to the number of photons incident on the cells surface at any wavelength *λ*. It is given as follows:

2 $$ \mathrm{IPCE}\%\left(\lambda \right)=1240\times \frac{J_{\mathrm{SC}}}{P_{in}\lambda } $$

IPCE values are also related to LHE, φE1, and ηEC. As shown in Eq. 3 [45],

3 $$ \mathrm{IPCE}\ \left(\lambda, \mathrm{nm}\right)=\mathrm{LHE}\upvarphi \mathrm{E}1\upeta \mathrm{E}\mathrm{C} $$

where LHE is the light harvesting efficiency, φE1 is electron injection quantum efficiency, and ηEC is the efficiency of collecting electrons in the external circuit.

## Limitations of the Devices

In the recent years, comparable efficiencies have been demonstrated for the DSSCs, but still they need a further modification due to some of the limitations associated with these cells. In terms of limitations, stability failure can be characterized in two different classes: (i) limitation towards extrinsic stability and (ii) limitation towards intrinsic stability. Also, a huge amount of loss in energy of oxidized dye takes place during the process of regeneration, due to the energy mismatch between the oxidized dye and an electrolyte. Thus, in the queue to enhance the efficacy of these cells, different electrolytes have been developed. Grätzel et al. showed over 900-mV open circuit voltages and short circuit currents *I*_SC_ up to 5.1 mA by blending the hole conductor matrix with a combination of TBP and Li[CF_3_SO_2_]_2_N, yielding an overall efficiency of 2.56% at air mass 1.5 (AM 1.5) illuminations [46]. Also, the sheet resistance of FTO glass sheet is about 10 Ω/sq.; thus, this makes scaling of the device difficult and acts as a limiting factor for an active cell area > 1 cm^2^. Therefore, to increase the sheet resistance as well as to maintain spacing between working and counter electrode, the short circuiting of the solar cell is required or either the spacing should be increased by 25 to 50 μm [47] in small modules (where these modules consist of small stripes of an active cell area (1 cm^2^) with adjacent silver lines [47, 48]). As a consequence, a drop in the IPCE value from 10.4 to 6.3% for a 1-cm^2^ cell was observed for a submodule of 26.5 cm^2^ [49].

To upscale the cell performance, silver fingers can be used to collect the current and using a sealant material like hotmelt tape, for the protection from the leakage of the electrolytes. Although due to the chemically aggressive nature of the electrolyte, the use of silver fingers is less feasible. And due to the small modules, the chances of leakage increase which results in reduced active cell area by 32% [47]. Another factor is the conductivity of the glass sheet that affects the performance of the DSSCs. Therefore, the conductivity of the transparent conducting oxide (TCO) can be improved by combining the indium-doped tin oxide (ITO, highly conductive but less chemically stable) and fluorine-doped tin oxide (FTO, highly chemically stable but less conductive) together. This results in the reduction of the sheet resistance of TCO glass to 1.3 Ω/sq. [50].

### Limitation Towards the Stability of the Devices

The DSSCs need to be stable extrinsically as well as intrinsically as to be comparable to that of Si-solar cells, so that they can fulfill market needs, and thus, their commercialization can be increased. The limitations towards the stability are discussed below:

#### Limitation Towards Extrinsic Stability (Stability of Sealant Material)

Sealant materials like Surlyn® and Bynel® hotmelt foils are used in DSSCs to seal the cells [48]. Their sealing capability decreases when the pressure builds up inside the cell [51] and also if exposed within a cyclic or regular temperature variation [52]. But due to their low cost and easy processing, their utilization cannot be neglected. Thus, it is required to increase their adhesion with glass by pretreatment of the glass with metal oxide particles. As an alternative, sealants based on low melting glass frits [53] were also developed which offer more stability than the hotmelt foils, but these sealants are not suitable for the large area module production.

#### Limitation Towards Intrinsic Stability

To examine the intrinsic stability of the cell, accelerated aging experiments were performed. These accelerated aging experiments lasts for 1000 h to show the thermal stability of the dye, electrolyte, and Pt-counter electrode at 80 °C of temperature. Through these experiments, it was found that small test cells can maintain 90% of the initial efficiency under elevated temperatures and the observed initial efficiencies were 7.65% [54] and 8% [55], respectively. Also, under AM 1.5 and 55–60 °C moderate temperatures, the device was stable for 1000 h. But when both the stress factors, i.e., temperature about 80 °C and light soaking, were combined, a rapid degradation in the performance of the cell was observed [52]. Therefore, improvement in the intrinsic stability of the cell is required as 80 °C temperature can be easily attained during sunny days.

## Different Ways to Augment the Efficiency of DSSCs

To enhance the efficiency as well as the stability of the DSSCs, researchers have to focus on fundamental fabrication methods and materials, as well working of these cells. Different ways to improve the efficiency of these solar cells (SCs) are discussed below:

1. To increase the efficiency of DSSCs, the oxidized dye must be firmly reduced to its original ground state after electron injection. In other words, the regeneration process (which occurs in the nanosecond range [56]) should be fast as compared to the process of oxidation of dye [the process of recombination (0.1 to 30 μs)]. As the redox mediator potential (I^−^ ion) strongly effects the maximum photovoltage, thus the potential of the redox couple should be close to the ground state of the dye. To carry out this viable repeated process, about 210 mV driving force is required (or ca. 0.6 V [56]).
2. By increasing the porosity of the TiO_2_ nanoparticles, the maximum dye absorption takes place at WE.
3. Reducing or prohibiting the formation of the dark current by depositing a uniform thin layer or under layer of the TiO_2_ nanoparticles over the conduction glass plate. Thus, the electrolyte does not have a direct contact with the FTO or back contact and hence not reduced by the collector electrons, which restricts the formation of the dark current.
4. Preventing the trapping of nanoporous TiO_2_ nanoparticles by TBP molecules or by an electrolyte solvent. Thus, uniform sensitization of the WE by a sensitizer is required. If the entire surface of the nanoporous TiO_2_ electrode is not uniformly covered by the sensitizer, then the naked spots of nanoporous TiO_2_ can be captured by TBP molecules or by an electrolyte solvent.
5. Co-sensitization is another way to optimize the performance of DSSC. In co-sensitization, two or more sensitizing dyes with different absorption spectrum ranges are mixed together. to broaden the spectrum response range [57].
6. By promoting the use of different materials in the manufacture of electrodes like nanotubes, nanowires of carbon, graphene; using varied electrolytes instead of a liquid one like gel electrolyte and quasi-solid electrolytes; providing different pre-post treatments to the working electrode like anodization pre-treatment and TiCl_4_ treatment; using different types of CEs [14] and by developing hydrophobic sensitizers, the performance as well as the efficiency of these cells can be tremendously improved.
7. By inserting phosphorescence or luminescent chromophores, such as applying rare-earth doped oxides into the DSSC [58–60], coating a luminescent layer on the glass of the photoanode [60–62], i.e., using plasmonic phenomenon [63] and adding energy relay dyes (ERDs) to the electrolyte [57, 64, 65].

## Previous and Further Improvements in DSSCs

To fabricate low cost, more flexible, and stable DSSCs with higher efficiencies, new materials that are light weight, thin, low cost, and easy to synthesize are required. Thus, previous as well as further improvement in the field of DSSCs is included in this section. This section gives a brief account on the work done by the different researchers in the last 10–12 years and the results they observed for respective cells.

### Working and Counter Electrodes

**Fig. 4:**
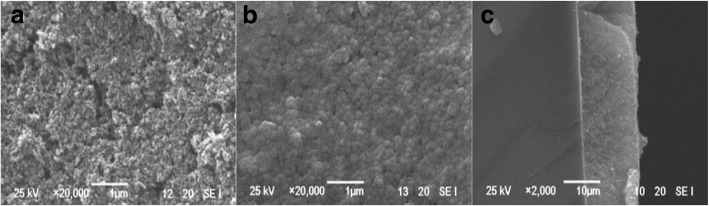
SEM images of **a** P25 film, **b** 1 wt% graphite-P25 composite film, and (**c**) the cross section of P25 film on FTO [69]

Grätzel and co-workers showed drastic improvements in the performance of DSSCs. They demonstrated efficiency of 7–10% under AM 1.5 irradiation using nanocrystalline (nc) TiO_2_ thin-film electrode with nanoporous structure and large surface area, and used a novel Ru bipyridyl complex as a sensitizer and an ionic redox electrolyte at EPFL [3, 26]. The conduction band level of TiO_2_ electrode and the redox potential of I^−^/I^−^ _3_ as − 0.7 V versus saturated calomel electrode (SCE) and 0.2 V versus SCE has been evaluated [66, 67]. A binary oxide photoelectrode with coffee as a natural dye was demonstrated, in 2014 [68]. SnO_2_ (*x*)–ZnO (1 − *x*) binary system with two different SnO_2_ composition (*x* = 3, 5 mol%) were prepared by solid-state reaction at high temperature and employed as a photoanode. An improved efficiency was demonstrated for the larger SnO_2_ composition and an overall power conversion efficiency (PCE) observed for SnO_2_: ZnO device was increased from 0.18% (3:97 mol%) to 0.26% for a device with SnO_2_:ZnO (5:95 mol%) photoanode. Hu et al. observed that the performance of the DSSCs with graphite-P25 composites as photoanodes has been significantly enhanced by 30% improvement of conversion efficiency compared with P25 alone. They found an enhancement in the value of *J*_SC_ from 9.03 to 12.59 mA/cm^2^ under the condition of 0.01 wt% graphite amount and attained the conversion efficiency of 5.76% [69]. Figure 4 shows the SEM images of the photoanodes. Apart from TiO_2_, carbon and its different allotropes are also widely applied in DSSCs to fulfill future demand and arisen as a perfect surrogate material for DSSCs. Some reports have shown that incorporating carbon nanotube (CNT) in TiO_2_ by hydrothermal or sol–gel methods greatly improved the cell’s performance [70–72]. Also, by improving the interconnectivity between the TiO_2_ and CNT, an increase in the IPCE can be found [70]. Sun et al. reported that the DSSCs incorporating graphene in TiO_2_ photoanode showed a PCE of 4.28%, which was 59% higher than that without graphene [73]. Sharma et al. has shown the improvement in the PCE value from 7.35 to 8.15% of the co-sensitized solar cell using modified TiO_2_ (G-TiO_2_) photoanode, instead of pure TiO_2_ photoanode [74]. In 2014, it was shown that the electronically and catalytically functional carbon cloth works as a permeable and flexible counter electrode for DSSC [75]. The researchers have found that the TiN nanotube arrays and TiN nanoparticles supported on carbon nanotubes showed high electrocatalytic activity for the reduction of triiodide ions in DSSCs [76, 77]. Single-crystal CoSe_2_ nanorods were applied as an efficient electrocatalyst for DSSCs by Sun et al. in 2014 [78]. They prepared single-crystal CoSe_2_ nanorods with a facile one step hydrothermal method. By drop-casting the CoSe_2_ nanorod suspension onto conductive substrates followed by simple drying without sintering, they fabricated the thin CoSe_2_ films and used as a highly efficient electrocatalyst for the reduction of I^−^ _3_. They showed a power conversion efficiency of 10.20% under AM1.5G one-sun illumination for DSSCs with the standard N719 dye. Park et al. prepared a mesoporous TiO_2_ Bragg stack templated by graft copolymer for dye-sensitized solar cells [79]. To enhance dye loading, electron transport, light harvesting and electrolyte pore-infiltration in DSSCs, they prepared organized mesoporous TiO_2_ Bragg stacks (om-TiO_2_ BS) consisting of alternating high and low refractive index organized mesoporous TiO_2_ (om-TiO_2_) films. They synthesized om-TiO_2_ films through sol-gel reaction using amphiphilic graft copolymers consisting of poly(vinyl chloride) backbones and poly(oxyethylene methacrylate) side chains, i.e., PVC-*g*-POEM as templates. They showed that a polymerized ionic liquid (PIL)-based DSSC fabricated with a 1.2-μm-thick om-TiO_2_ BS-based photoanode exhibited an efficiency of 4.3%, which was much higher than that of conventional DSSCs with a nanocrystalline TiO_2_ layer (nc-TiO_2_ layer) with an efficiency of 1.7%. An excellent efficiency of 7.5% was demonstrated for a polymerized ionic liquid (PIL)-based DSSC with a heterostructured photoanode consisting of 400-nm-thick organized mesoporous TiO_2_ interfacial (om-TiO_2_ IF) layer, 7-μm-thick nc-TiO_2_, and 1.2-μm-thick om-TiO_2_ BS as the bottom, middle, and top layers, respectively, which was again much higher than that of nanocrystalline TiO_2_ photoanode with an efficiency of 3.5%. Lee et al. reported platinum-free, low-cost, and flexible DSSCs using graphene film coated with a conducting polymer as a counter electrode [80]. In 2014, Banerjee et al. demonstrated nickel cobalt sulfide nanoneedle-array as an effective alternative to Pt as a counter electrode in dye-sensitized solar cells [81].

**Fig. 5:**
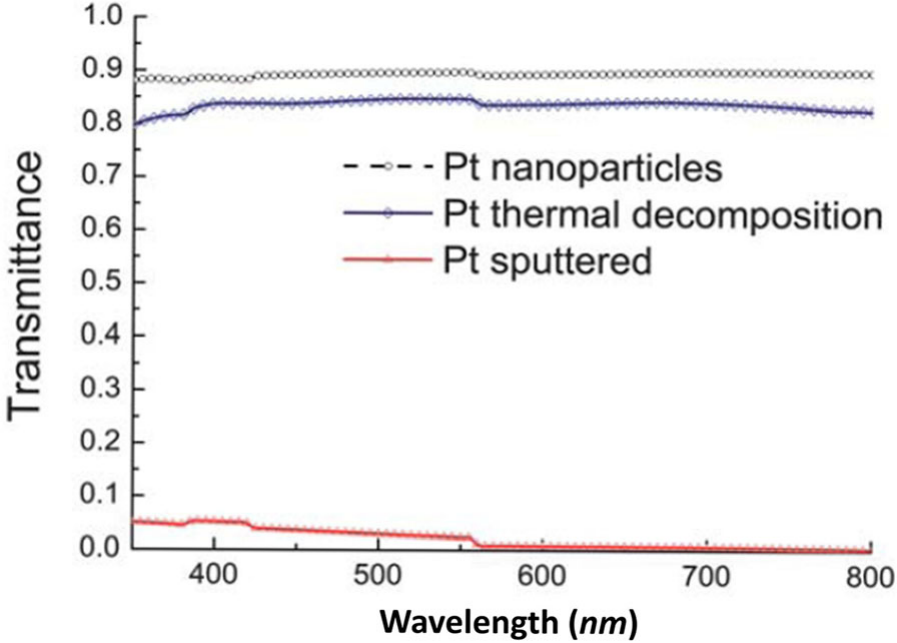
Optical transmittance of platinum-based films (Pt nanoparticles, Pt thermal decomposition, Pt sputtered) deposited onto FTO glass [82]

Calogero et al. invented a transparent and low-cost counter electrode based on platinum nanoparticles prepared by a bottom-up synthetic approach. They demonstrated that with such a type of cathode, the observed solar energy conversion efficiency was the same as that obtained for a platinum-sputtered counter electrode and even was more than 50% obtained with a standard electrode, i.e., one prepared by chlorine platinum acid thermal decomposition, in similar working condition [82]. By using a special back-reflecting layer of silver, they improved upon the performance of a counter electrode based on platinum sputtering and achieved an overall *η* of 4.75% under 100 mWcm^− 2^ (AM 1.5) of simulated sunlight. They showed that, for the optical transmittance at different wavelengths of platinum-based films, i.e., Pt nanoparticles, Pt thermal decomposition, and Pt sputtered deposited onto FTO glass, the platinum nanoparticle-based cathode electrode (CE) prepared by Pt sputtering deposition method appeared more transparent than the platinum CE prepared using the Pt acid thermal decomposition method. Meanwhile, when Pt nanoparticle deposition method was employed, the transmittance was very poor (as shown in Fig. 5). Anothumakkool et al. showed a highly conducting 1-D aligned polyethylenedioxythiophene (PEDOT) along the inner and outer surfaces of a hollow carbon nanofiber (CNF), as a counter electrode in a DSSC to enhance the electrocatalytic activity of the cell [83]. They showed that the hybrid material (CP-25) displayed a conversion efficiency of 7.16% compared to 7.30% for the standard Pt counter electrode, 4.48% for bulk PEDOT and 5.56% for CNF, respectively. The enhanced conversion efficiency of CP-25 was accredited to the accomplishment of high conductivity and surface area of PEDOT through the 1-D alignment compared to its bulk counterpart. Further, through a long-term stability test involving efficiency profiling for 20 days, it was observed that CP-25 exhibited extraordinary durability compared to the bulk PEDOT. Recently, Huang et al. improved the performance of the device by inserting a H_3_PW_12_O_40_ layer between the transparent conductive oxide layer and the compact TiO_2_ layer [84]. They observed the reduction in the recombination of the electrons upon the addition of H_3_PW_12_O_40_ layer, resulting in longer electron lifetime and obtained a *η* = 9.3%, respectively.

**Fig. 6:**
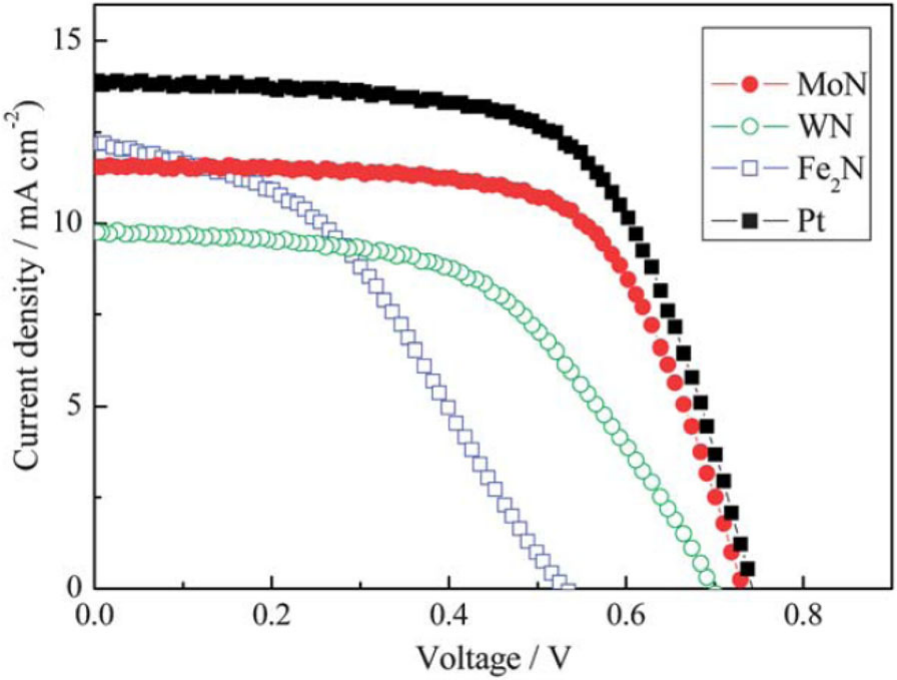
Characteristic *J*–*V* curves of DSSCs using different metal nitrides and Pt counter electrodes, measured under simulated sunlight at 100 mWcm^− 2^ (AM 1.5) [85]

Li et al. reported that the transition metal nitrides MoN, WN, and Fe_2_N show Pt-like electrocatalytic activity for dye-sensitized solar cells, where MoN showed superior electrocatalytic activity and a higher PV performance [85]. Characteristic *J–V* curves of DSSCs using different metal nitrides and Pt counter electrodes showed that the cell fabricated with the MoN counter electrode achieved a FF = 0.66, which was higher than that of the Pt electrode (as shown in Fig. 6). However, *J*_SC_ = 11.55 mAcm^− 2^ was relatively high and the *V*_OC_ of 0.735 V was almost same to the *V*_OC_ = 0.740 V offered by Pt electrode. In the case of WN, *V*_OC_ and *J*_SC_ were relatively low, indicating a low efficiency of 3.67%. DSSC with the Fe_2_N electrode attained lower values of *V*_OC_ and FF, i.e., 0.535 V of *V*_OC_ and 0.41 of FF, resulting in a poor *η* = 2.65%. Thus, above data shows superior performance of MoN-based DSSC among all other metal nitrides as CE material. Gokhale et al. showed a laser-synthesized super-hydrophobic conducting carbon with broccoli-type morphology as a CE for dye-sensitized solar cells in 2012 [86]. In 2014, plasmonic light harvesting of dye-sensitized solar cells by Au nanoparticle-loaded TiO_2_ nanofibers was demonstrated by Naphade et al. [87] because the surface morphology of a WE and a CE play a key role in the performance of DSSC. Usually, mesoporous TiO_2_ nanoparticle films are used in WE fabrication because they provide large surface area for efficient dye adsorption. However, there are certain limitations associated with them as short electron diffusion length (10–35 μm) and random electrical pathway induced by the substantial trapping and detrapping phenomena that take place within excessive surface states, defects, and grain boundaries of nanoparticles [88] and disorganized stacking of TiO_2_ films which limits the electron transport [89]. Thus, doping of metallic cations and non-metallic anions in TiO_2_, treating FTOs [90], applying 1-D nanostructures like nanowires, nanorods, nanosheets, nanoplates [16], and hollow spheres are approaches to modify the WE. However due to the low surface area, these 1-D nanostructures show poor dye loading. In 2015, Zhao et al. studied the influence of the incorporation of CNT-G-TiO_2_ NPs into TiO_2_ NT arrays and attained an efficiency of 6.17% for the DSSC based on CNT-G-TiO_2_ nanoparticles/TiO_2_ nanotube double-layer structure photoanode [91]. An efficiency of 8.30% was demonstrated by Qiu et al. for the DSSC based on double-layered anatase TiO_2_ nanospindle photoanodes [92].

Apart from NTs, bilayer TiO_2_ hollow spheres/TiO_2_ nanotube array-based DSSC also showed an effective efficiency of 6.90% [93]. Efficiency can also be improved by incorporating SnO_2_ as a shell material on a photoanode [94]. The integration of SnO_2_ as a shell material on ZnO nanoneedle arrays results in a larger surface area and reduced recombination rate [94], thus increasing the dye adsorption which plays a crucial role in the performance of a cell. Huang and co-workers synthesized mesoporous TiO_2_ spheres of high crystallinity and large surface area and applied it as a WE in the device. An excellent efficiency of 10.3% was achieved for the DSSC-employed TiO_2_ spheres with long-term stability due to the terrific dye-loading and light-scattering abilities as well as attenuated charge recombination. Further, the efficiency was improved by performing the TiCl_4_ treatment [95].

Maheswari et al. reported various DSSCs employing zirconia-doped TiO_2_ nanoparticle and nanowire composite photoanode film. They demonstrated highest *η* = 9.93% for Zirconia/TNPW photoanode with a hafnium oxide (HfO_2_) blocking layer and observed that the combination of zirconia-doped photoanode with blocking layer possibly restrains the recombination process and increases the PCE of the DSSCs effectively [96]. However, many ideas do not achieve a great efficiency initially but at least embed different ideas and aspects for the synthesis of new materials. For instance, by using carbon-coated stainless steel as a CE for DSSC, Shejale et al. demonstrated a *η* = 1.98%, respectively [97]. Recently in 2018, a study was carried out to determine the effect of microwave exposure on photoanode and found an enhancement in the efficiency of the cell upon exposure. For the preparation of the DSSC, a LiI electrolyte, Pt cathode, TiO_2_ photoanode, and Alizarin red as a natural sensitizer were used. An efficiency of 0.144% was found for the cell, where 10 min of microwave exposure was carried upon the photoanode [98].

**Fig. 7:**
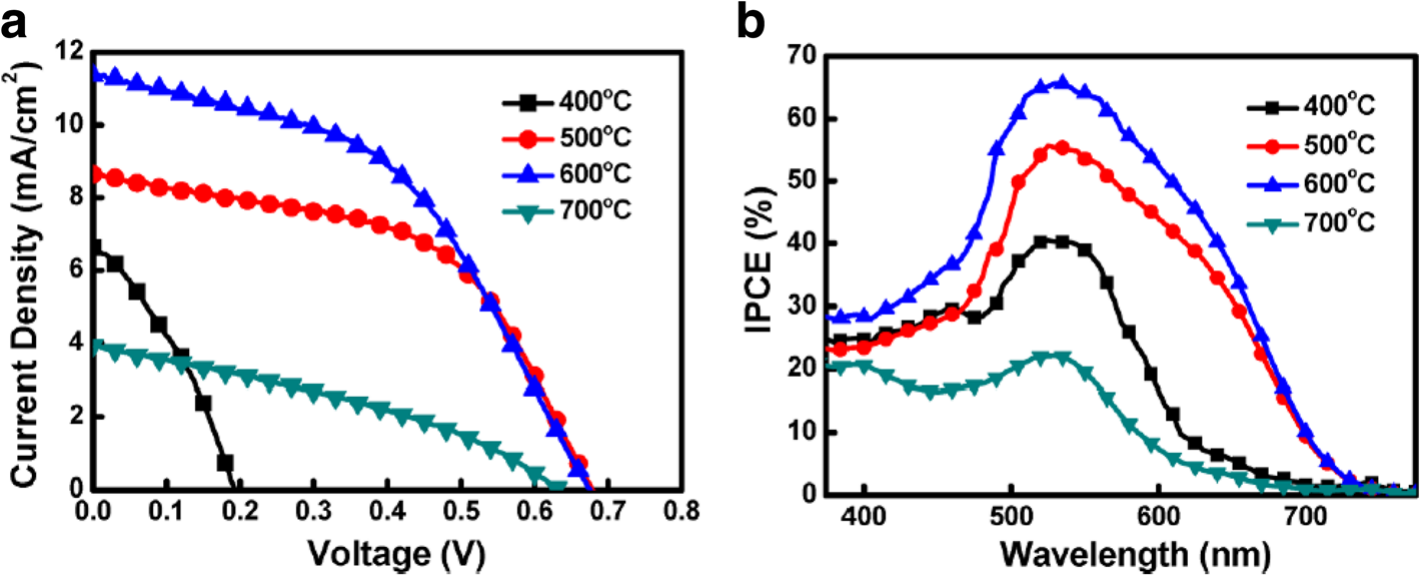
The **a** current density–voltage (*J*–*V*) and **b** incident monochromatic photon-to-current conversion efficiency (IPCE) curves of DSSCs using various Cu_2_O CE [102]

**Fig. 8:**
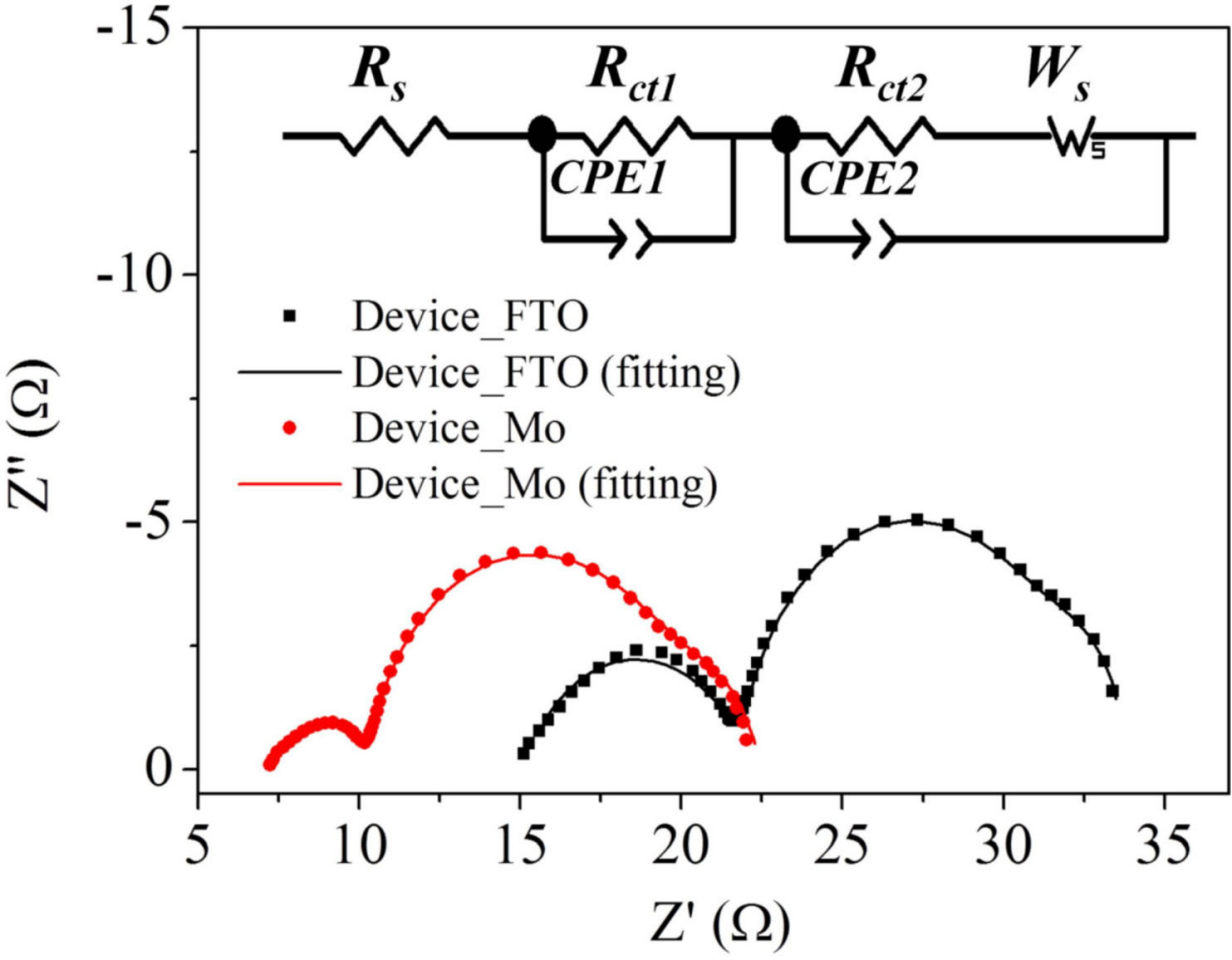
Nyquist plots of the Device_FTO and Device_Mo. The inset indicates an equivalent circuit model used for the devices [103]

**Fig. 9:**
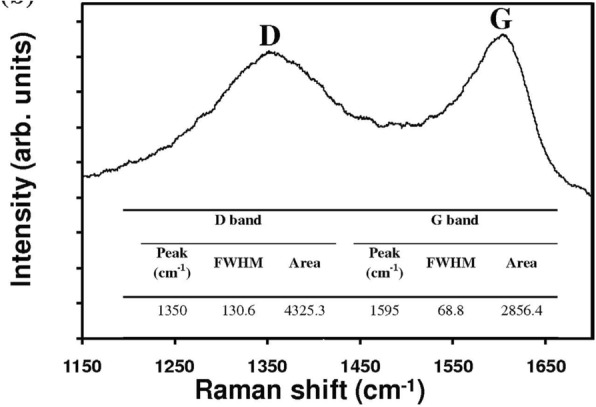
Raman spectra of mangosteen peel carbon [104]

Similarly, varied materials as mentioned earlier are synthesized as CE for efficient DSSCs. Last year, Guo et al. synthesized an In_2.77_S_4_@conductive carbon (In_2.77S4_@CC) hybrid CE via a two-step method and achieved *η* = 8.71% for the DSSC with superior electrocatalytic activity for the reduction of triiodide and, also, comparable to the commercial Pt-based DSSC that showed PCE of 8.75%, respectively [99]. The doping of an organic acid, 1S-(+)-camphorsulfonic acid, with the conductive polymer poly(o-methoxyaniline) to form a hybrid (CSA/POMA) and its application in DSSCs as CE has been examined by Tsai et al. This CE showed increased surface roughness, decreased impedance, and increased crystallinity [100]. In 2017, Liu et al. fabricated DSSCs employing Co(bpy)_3_^3+/2+^ as the redox couple and carbon black (CB) as the CE [101]. The observation revealed superior electrocatalytic activity of a well-prepared CB film compared to that of conventional sputtered Pt. Due to the flexible nature of Cu foil substrates, Cu_2_O has also been employed as a CE in DSSC [102]. The fabrication of different samples by varying the sintering temperature of the CEs and obtaining the maximum efficiency of 3.62% at 600 °C of temperature has been reported [102]. Figure 7 shows the *I*–*V* characteristics and IPCE curves of DSSCs employing different Cu_2_O CEs. In 2013, by replacing the FTO with Mo as the conductor for the counter electrode, an increase in the value of FF as well as *η* was found [103]. The EIS Nyquist plots (as shown in Fig. 8) showed the difference in *R*_s_ between the devices employed FTO (15.11 Ωcm^2^) and Mo (7.25 Ωcm^2^) due to the dissimilarity of the sheet resistance between FTO (8.2 Ω/sq) and Mo (0.16 Ω/sq). Also, by replacing FTO with Mo, a decrease in the *R*_ct1_ value from 6.87 to 3.14 Ωcm^2^ was induced by the higher redox reactivity of Pt on Mo than that on FTO. In the queue of developing new materials, Maiaugree et al. fabricated DSSCs employing carbonized mangosteen peel (MPC) as a natural counter electrode with a mangosteen peel dye as a sensitizer [104]. They observed a typical mesoporous honeycomb-like carbon structure with a rough nanoscale surface in carbonized mangosteen peels and achieved the highest value of *η* = 2.63%. By analyzing the Raman spectra (shown in Fig. 9), they found a broad D-peak (130.6 cm^− 1^ of FWHM) located at 1350 cm^− 1^ indicating the high disorder of sp3 carbon and a narrower G peak (68.8 cm^− 1^ of FWHM) at 1595 cm^− 1^ which correlated with a graphite oxide phase observed in 2008 [105]. Thus, it was concluded the graphite oxide from MPC was a highly ordered sp2 hexagonal carbon oxide network. Furthermore, *I–V* characteristics of DSSCs employing different WE and CE are summarized in Table 1.

**Table 1:** Photovoltaic parameters of DSSCs employing different types of WEs and CEs

WE/CE	*V*_OC_ (mV)	*J*_SC_ (mAcm^− 2^)	FF (%)	*η* (%)	Reference
WE: TiO_2_ doped with tungsten	730	15.10	67	7.42	[328]
WE: TiO_2_ doped with scandium	752	19.10	68	9.60	[329]
WE: TiO_2_ doped with indium	716	16.97	61	7.48	[330]
WE: TiO_2_ doped with boron	660	7.85	66	3.44	[331]
WE: TiO_2_ doped with fluorine	754	11	76	6.31	[332]
WE: TiO_2_ doped with carbon	730	20.38	57	8.55	[333]
WE: ONT/FTO	700	10.65	70	5.32	[334]
WE: G-TiO_2_ NPs/TiO_2_ NTs	690	16.59	56	6.29	[335]
WE: TiO_2_ doped with Cu	591	6.84	56	2.28	[336]
WE: 7.5% SnO_2_ doped TiO_2_	790	14.53	58	6.7	[337]
WE: TiO_2_:Y_1.86_Eu_0.14_WO_6_	757	12.3	43	3.9	[338]
WE: Nb2O5	738	6.23	68.3	3.15	[339]
WE: Nanographite-TiO_2_	720	1.69	35	0.44	[340]
CE: PtCo	717	16.96	66	7.64	[341]
CE:Pt+SLGO	670	7.9	65	3.4	[342]
CE: PtMo	697	15.48	62	6.75	[343]
CE: PtCr_0.05_	739	13.07	71	6.88	[344]
CE: CoNi_0.25_	706	18.02	66	8.39	[42]
CE: Ni-PANI-G	719	11.56	64	5.32	[345]
CE: PANI nanoribbons	720	17.92	56	7.23	[346]
CE: Pd_17_Se_15_	700	16.32	65	7.45	[347]
CE: PtCuNi	758	18.30	69	9.66	[348]
CE: g-C_3_N_4_/G	723	14.91	66	7.13	[349]
CE: FeN/N-doped graphene	740	18.83	78	10.86	[350]
CE: MoS_2_ nanofilm	740	16.96	66	8.28	[351]
CE: Ni_0.33_Co_0.67_Se microsphere	789	17.29	67	9.01	[352]
CE: Tubular orthorhombic CoSe_2_	771	17.35	70	9.34	[353]
CE: CoSe_2_	809	17.65	71	10.17	[354]
CE: In_2.77_S_4_@CC	750	17.34	67	8.71	[99]
CE: Electrochemically deposited Pt	750	17.16	60	7.72	[355]
CE: Fe_3_O_4_@RGO-NMCC	760	17.00	70	9.04	[356]
CE: CB-NPs/s-PT	764	17.21	69	9.02	[357]
CE: AC/MWCNTs	753	16.07	83	10.05	[358]
CE: NiCo_2_S_4_	148.6	2.98	55.8	0.24	[359]
CE: SS:Graphene	524	1.46	26	1.98	[97]
CE: Ru0.33Se	722	17.86	67.9	8.76	[360]
CE:5% Ag-doped SnS_2_	740	16.7	70	8.70	[361]
CE: Cu_2_O	680	11.35	47	3.62	[102]

### Electrolyte

To improve and study the performance of DSSCs, different electrolytes like gel electrolytes, quasi-solid-state electrolytes, ionic liquid electrolytes etc. have been applied as mediators so far. However, a different trend to optimize the performance of the DSSCs has been initiated by adding the energy relay dyes to the electrolyte.

#### Liquid Electrolyte

The cells efficiency through liquid electrolyte can be augmented by introducing iodide/triiodide redox couple and high dielectric constant organic solvents like ACN, 3-methoxypropionitrile (MePN), propylene carbonate (PC), γ-butyrolactone (GBL), *N*-methyl-2-pyrrolidone (NMP), ethylene carbonate (EC), and counter ions of iodides, where solvents are the key component of a liquid electrolyte. On the basis of their stability, organic solvents can be sequenced as imidazolium < picolinium < alkylpyridinium. Among various characteristics of solvents like donor number, dielectric constants, and viscosity, the donor number shows manifest influence on the *V*_OC_ and *J*_SC_ of DSSCs. Adding the small amount of electric additives like *N*-methylbenzimidazole (NMBI), guanidinium thiocyanate (GuSCN), and TBP hugely improves the cell performance. Just like solvents, a coabsorbent also plays a key role in the functioning and performance of an electrolyte. The addition of coabsorbents in an electrolyte trims down the charge recombination of photoelectrons in the semiconductor with the redox shuttle of the electrolyte. Secondly, a coabsorbent may alter the band edge position of the TiO_2_-conduction band, thus resulting in an augmentation in the value of *V*_OC_ of the cell. This suppresses the dye aggregation over the TiO_2_ surface and results in long-term stability of the cell as well as increase in *V*_OC_. Although the best regeneration of the oxidized dye is observed for iodide/triiodide as a redox couple for a liquid electrolyte, its characteristic of severe corrosion for many sealing materials results in a poor long-term stability of the DSSC. Thus SCN^−^/SCN_2_, Br^−^/Br_2_, and SeCN^−^/SeCN_2_ bipyridine cobalt (III/II) complexes are some of the other redox couples applied in DSSCs. The ionic liquids (IL) or room temperature ionic liquids (RTIL) are stand-in material for organic solvents in a liquid electrolyte. Despite the many advantages, i.e., negligible vapor pressure, low flammability, high electrical conductivity at room temperature (RT), and wide electrochemical window, they are less applicable in DSSCs. Because of their higher viscosity, restoration of oxidized dye restricts due to the lower transport speed of iodide/triioide in solvent-free IL electrolytes. Thus, the performance of the dye-sensitized solar cells can also be enhanced by modifying the TiO_2_ dye interface, i.e., by reducing vapor pressure of the electrolyte’s solvent. In 2017, Puspitasari et al. investigated the effect of mixing dyes and solvent in electrolyte and thus fabricated various devices. They have used two types of gel electrolyte based on PEG that mixed with liquid electrolyte for analyzing the lifetime of DSSC. They also changed solvents as distilled water (type I) and ACN (type II) with the addition of concentration of KI and iodine, and achieved better efficiency for the electrolyte type II [106].

As low-viscous solution can cause leakage in the cell, thus, application of solidified electrolytes obtained by in situ polymerization of precursor solution containing monomer or oligomer and the iodide/iodine redox couple results in a completely filled quasi-solid-state electrolyte within the TiO_2_ network with negligible vapor pressure [107]. Komiya et al. obtained initial efficiency of 8.1% by applying the aforementioned approach [107]. But still a question arises whether the polymer matrix will degrade under prolonged UV radiations or not. The effect on the addition of SiO_2_ nanoparticles to solidify the solvent was also studied as to increase the cell efficiency [108], where only inorganic materials were applied in this technique. However, there are certain limitations associated with the addition of organic solvents within a liquid electrolyte, i.e., this leads hermetic sealing of the cell and the evaporation of solvents at higher temperature, and thus the cells do not uphold long-term stability. Therefore, more research was carried over the developments and implementation of gel, polymer, and solid-state electrolytes in the DSSCs with various approaches, such as the usages of the electrolytes containing p-type inorganic semiconductors [109], organic hole transporting materials (HTMs) [110], and polymer gelator (PG) [111]. Chen et al. fabricated a solid-state DSSC using PVB-SPE (polyvinyl butyral-quasi-solid polymeric electrolyte) as an electrolyte. They measured the efficiency approximately 5.46%, which was approximately 94% compared to that of corresponding liquid-state devices, and the lifetime observed for the devices was over 3000 h [112]. Recently, a study explained the stability of the current characteristics of DSSCs in the second quadrant of the *I–V* characteristics [113]. The study explains the continuous flow of the forward current and the operating voltage point that gradually shift towards more negative voltages in the second quadrant of the *I–V* characteristics. The increase in the ratio of iodide to tri-iodide in the electrolyte rather than to the decomposition or the coupling reactions of the constituent materials was considered to be the reason behind it. According to the studies, these changes were also considered as reversible reactions that can be detected based on the changes in the color of the electrolyte or the *I–V* measurements.

However, ILs with lower viscosity and higher iodine concentration are needed as to increase *J*_SC_ by increasing iodine mass transport. Laser transient measurements have been attempted and revealed that the high iodide concentration present in the pure ILs leads to a reductive quenching of the excited dye molecule [114]. Due to the low cost, thermal stability, and good conductivity of the conductive polymers based on polytiophenes and polypyrroles, they can be widely applied in DSSCs despite using ILs [115]. For the application point of view, the IL should have a high number of delocalized negative charge and counterions with a high chemical stability. Also, the derivatives of imidazolium salts are one of the best applicable in DSSCs. When 1-ethyl-3-methylimidazolium dicyanamide [EMIM] [DCA] with a viscosity of only 21 mPa s [116] was combined with 1-propyl-3-methylimidayolium iodide (PMII, volume ratio 1:1), an efficiency of 7.4% was observed and, after prolonged illumination, some degradation was also found. A cell with a binary IL of 1-ethyl-3-methylimidazolium tetracyanoborate in combination with PMII showed a stable efficiency of 7% that retained at least 90% of its initial efficiency after 1000 h at 80 °C in darkness and 1000 h at 60 °C, at AM 1.5 [117]. Moudam et al. studied the effect of water-based electrolytes in DSSC and demonstrated a highly efficient glass and printable flexible dye-sensitized solar cells upon application [118]. They used high concentrations of alkylamidazoliums to overcome the deleterious effect of water. The DSSCs employed pure water-based electrolyte and were tested under a simulated air mass 1.5 solar spectrum illumination at 100 mWcm^− 2^ and found the highest recorded efficiency of 3.45% and 6% for flexible and glass cells, respectively. An increase in the value of *V*_OC_ from 0.38 to 0.72 V on the addition of TBP to the electrolyte has been observed [26]. Thus, to improve the efficiencies of DSSC, new materials were synthesized and applied in DSSCs. L-cysteine/L-cystine redox couple was employed in DSSC by Chen et al. which showed a comparable efficiency of 7.70%, as compared to the cell using I^−^/I_3_^−^ redox couple (8.10%) [119]. In 2016, Huang et al. studied the effect of liquid crystals (LCs) on the PCE of dye-sensitized solar cells. They observed that the addition of minute amounts of LC decreases the *J*_SC_ because it reduces the electrochemical reaction rate between the counter electrode and an electrolyte. Also, it delays the degradation rates of the cell because of the interaction between cyano groups of the doped LCs and organic solvent in the liquid electrolyte [120]. Main components of different kinds of electrolytes are discussed below:

##### Pyridine Derivatives (Like 4-Tert-Butylpyridine [TBP], 2-Propylpyridine, *N*-Methylbenzimidazole [NMBI])

The improved efficiency for a DSSC can be achieved by adding about 0.5 M of pyridine derivative within the electrolyte, due to which an increase in the value of *V*_OC_ occurs. This improved *V*_OC_ can be attributed to the positive band edge movement and slightly affected charge recombination rate on the basis of intensity-modulated photovoltage spectroscopy (IMVS) [121]. The study showed that after the adsorption of pyridine ring on TiO_2_ surface, the pyridine ring induced electron density into the TiO_2_ creating a surface dipole. But, the band edge movement results in the slight decrease in *J*_SC_ as compared to the untreated cell, due to the diminution in the driving force for electron injection [122]. Further, application of NMBI over TBP was studied in 2003, due to its long-term stability under elevated temperature [54].

##### Alkyl Phosphonic/Carboxylic Acids (Like Decylphosphonic Acid [DPA], Hexadecylmalonic Acid [HDMA])

An improved *V*_OC_ with slight decrease in the *J*_SC_ have been observed when DPA [54] and HDMA [123] were combined. This was due to the presence of self-assembled long alkyl chain on the surface of TiO_2_, which is responsible for the formation of densely packed hydrophobic monolayer and reduction in recombination rate too, as these long alkyl chains repel iodide from TiO_2_ surface.

##### Guanidinium Derivatives (GuSCN)

The addition of guanidium thiocyanate as a co-absorbent in an electrolyte results in enhanced *V*_OC_ by ca. 120 mV with a downward shift in the conduction band by ca. 100 mV [124] at the same time due to the suppression in the recombination rate by a factor of 20 and a difference of 20 mV gained for *V*_OC_. By limiting the downward shift in the conduction band, an improvement in the overall efficacy can be attained.

#### Solid-State Electrolyte (SSE)

The SSE falls in two subcategories: (1) where hole transport materials are used as a transport medium and (2) SSE containing iodide/triiodide redox couple as a transport medium. Both kinds of SSEs are discussed below:

##### Hole Transport Materials (HTMs)

HTMs fall in the category of solid-state electrolytes, where HTMs are used a medium. These materials have set a great milestone in DSSCs and effectively applicable in cells because iodine/iodide electrolytes are highly chemically aggressive by nature and corrodes other materials easily, mostly metals. Most of the HTMs are chemically less-aggressive inorganic solids, organic polymers, or p-conducting molecules, although the results are still unmatched with the one obtained for iodine/iodide redox electrolytes because of the following reasons:

1. Due to their solid form, an incomplete penetration of solid HTMs within nanoporous TiO_2_-layer leads to poor electronic contact between HTMs and the dye. Thus, incomplete dye regeneration takes place.
2. The high frequencies of charge recombination from TiO_2_ to HTMs.
3. Due to the presence of organic hole conducting molecules, the series resistance of the cell increases due to the low hole mobility in the organic HTMs as compared to IL electrolytes.
4. HTM results in a drop in *V*_OC_, as the recombination rate of electrons of CB with HTM becomes higher as compared to iodine/iodide redox electrolytes.
5. Low intrinsic conductivities of HTMs.

Thus, researchers need to synthesize and focus on HTMs whose VB energy should be slightly above the energy of the oxidized dye, should not absorb light, and must be photochemically stable, so that they can keep a healthy contact with the dye. Among a number of HTMs, some of the HTMs are discussed below:

##### Inorganic CuI Salt

CuI halogens and pseudohalogens are two classes of inorganic CuI salts that can be applied as HTMs in a DSSC. Copper bromide (CuBr), copper iodide (CuI), and copper thiocyanate (CuSCN) are some copper-based compounds which work as a hole conductor and are more effective due to their good conductivity. Although CuSCN is one of the best pseudohalogen HTMs and despite its high hole mobility, its application results in high series resistance and does not support high current and also shows poor electronic contact between CuSCN and the dye, and poor pore filling due to their fast crystallization rates, which resulted in low *η* of < 4% for the corresponding solid-state DSSCs [125]. Thus, to reduce the high recombination rate of electrons, additional blocking layers of insulating materials like SiO_2_ or Al_2_O_3_ can be applied or coated around the TiO_2_ particles which enhance the *V*_OC_ due to the suppressed recombination rate. With respect to halogens, CuBr showed an efficiency of 1.53% with thioether as an additive [126] and demonstrated high stability under prolonged irradiation of about 200 h at RT and the application of nickel oxide (NiO) showed moderate PCEs of 3% [127]. But, due to the easy poor solubility as well as crystallization of these materials, their application became a challenge and, thus, pseudohalogens have proven to be more stable and efficient in DSSCs. But the devices were found to be highly unstable and the reproducibility became dubious.

##### Hole-Conducting Molecules

spiro-OMeTAD {2,2′,7,7′-tetrakis(N,N′-di-p methoxyphenylamine)-9,9′-spirobifluorene} is one of the most suitable candidate in the prospect of hole conducting molecules and thus also widely applicable in integrated devices [128]. It was first introduced in 1998 [110] with a high glass transition temperature of ca. 120 °C. The researchers observed the formation of amorphous layers that are necessary for the complete pore filling and showed an IPCE of 33%, yielding overall efficiency to about 0.74% [110], and finally 4% of efficiency with an ambiphillic dye Z907 was demonstrated [129]. Some other triphenylamine detrivatives also demonstrated sufficient efficiencies in DSSCs [130]. Again, spiro-OMeTAD has certain limitations as it has low charge carrier mobility, ca. 104 cm^2^/Vs [130], that limits the thickness of the TiO_2_ layer up to 2 μm and thus leads to incomplete light harvesting efficiency (LHE) of dye. Also, a high recombination rate between TiO_2_ and FTO leads to low efficiencies in DSSCs.

##### Triphenylamine (TPA)

Phenylamines demonstrate a remarkable charge transporting property which makes them great hole transporting materials in organic electroluminescent devices [131]. However, despite a huge range of non-conjugated polymers of di- and triphenylamine which are synthesized and used efficiently as HTMs in organic electroluminescent devices, their conjugated polymers are still rare. Polyaniline (PANI) is the only well-recognized conjugated diphenylamine polymer [132] due to its highly electrical conductive property and is environmentally stable in the doped state. In 1991, triphenylamine (TPA)-conjugated polymers were synthesized by Ni-catalyzed coupling polymerization [133]. Okada et al. reported dimer (TPD 9), trimer (TPTR 10), tetramer (TPTE 11), and pentamer (TPPE 12) of TPA with the aid of Ullmann coupling reaction between the corresponding primary or secondary arylamines and aryl iodides [134].

##### SSE Containing Iodide/Triiodide Redox Couple

These SSEs have larger applications than those of HTMs, because interfacial contact properties of these solid-state electrolytes are better than those of HTMs. Fabrication of a DSSC based on solid-state electrolyte was reported by adding TiO_2_ nanoparticle into poly(ethylene oxide) (PEO) and the overall light-to-electricity conversion efficiency of 4.2% for the cell was obtained under irradiation of AM 1.5100 mWcm^− 2^ [135].

#### Quasi-Solid-State Electrolyte (QSSE)

**Fig. 10:**
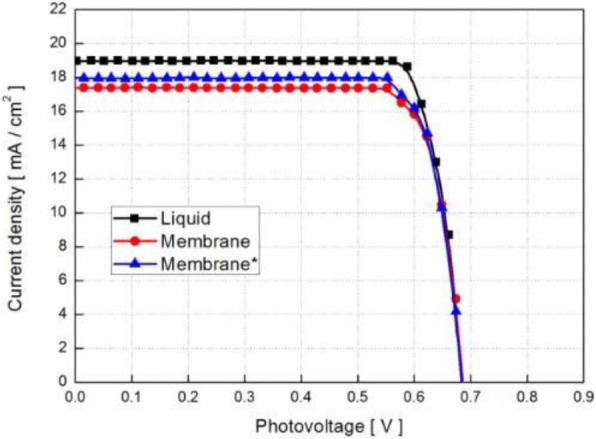
Polarization curves of DSSCs with various electrolytes under simulated AM 1.5 global sunlight (1 sun, 100 mWcm^−2^) [138]

QSSE has a hybrid network structure, because it consists of a polymer host network swollen with liquid electrolytes, thus showing the property of both solid (cohesive property) and liquid (diffusive transport property), simultaneously. Thus, to overcome the volatilization and leakage problems of liquid electrolytes, ILs like 1-propargyl-3- methylimidazolium iodide, bis(imidazolium) iodides and 1-ethyl-1-methylpyrrolidinium and polymer gel-like PEO, and poly(vinylidinefluoride) and polyvinyl acetate containing redox couples are commonly used as QSSEs [136, 137]. In 2015, Sun et al. fabricated a DSSC employing wet-laid polyethylene terephthalate (PET) membrane electrolyte, where PET is a commonly used textile fiber used in the form of a wet-laid non-woven fabric as a matrix for an electrolyte. According to their observation, this membrane can better absorb electrolyte turning into a quasi-solid, providing excellent interfacial contact between both electrodes of the DSSC and preventing a short circuit. The quasi-solid-state DSSC assembled with an optimized membrane exhibited a PCE = 10.248% at 100 mWcm^−2^. To improve the absorbance, they plasma-treated the membrane separately with argon and oxygen, which resulted in the retention of the electrolyte, avoiding its evaporation, and a 15% longer lifetime of the DSSC compared to liquid electrolyte [138]. Figure 10 shows the polarization curves of DSSCs with various electrolytes under simulated AM 1.5 global sunlight (1 Sun, 100 mWcm^−2^).

##### Hole-Conducting Polymers

**Fig. 11:**
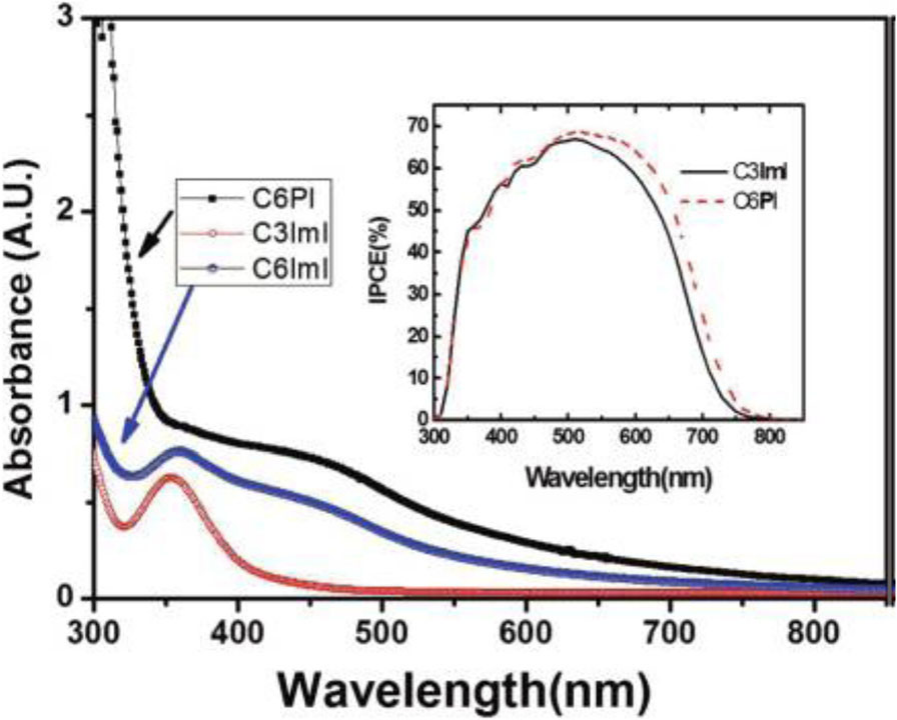
UV-vis spectroscopy selected pyridinium and imidazolium salts. The inset is the IPCE data for the cells with EC3ImI and EC6PI, which are the best cells among each series [141]

IPCE of 3.5% by the application of C60/polythiphene derivative in DSSCs has been achieved for pure organic solar cells [139]. However, this field is developing slowly, as its deposition by standard methods (like CBD) is difficult, because solid polymer does not penetrate the TiO_2_-nanoporous layer. Hence, there are only few groups applied as conducting polymers in DSSCs. Ravirajan et al. demonstrated a monochromatic efficiency of 1.4% at 440 nm by applying fluorene-thiophene copolymer [140]. Researchers are working hard so long to develop new efficient materials for electrolytes. Jeon et al. reported that the addition of alkylpyridinium iodide salts in electrolytes enhanced the performance of the dye-sensitized solar cells. They observed better *J–V* characteristics, 7.92% efficiency with *V*_OC_ = 0.696 V, *J*_SC_ = 17.74 mA/cm^2^, and FF = 0.641 for the cell applying EC6PI (pyridinium salts) as compared to EC3ImI (imidazolium salts), whose *η* = 7.46% with *V*_OC_ = 0.686 V, *J*_SC_ = 16.99 mA/cm^2^, and FF = 0.64 [141]. For a comparison, they added UV spectra for C6ImI and observed that the higher quantum efficiencies from the cell with EC6PI were obtained within the wide range from 460 to 800 nm. The quantum efficiencies were almost the same in the range of shorter wavelengths, may be due to the ability of C6PI to absorb more incident light than C3ImI at shorter wavelengths. Even so, the absorption coefficients for C6PI were higher than those for C6ImI over all the range, but the cell efficiencies are quite comparable (as shown in Fig. 11) [141]. Lee et al. developed and utilized the conjugated polymer electrolytes (CPEs) like MPF-E, MPCZ-E, MPCF-E, and MPCT-E containing quaternized ammonium iodide groups in polymer solution and gel electrolytes for DSSCs. They observed, as the polymer content in the electrolyte solution increased, the electrochemical impedance also increased for the cells based on CPE containing polymer solution electrolytes, whereas the PV performances showed the reverse trend [142]. Table 2 shows the FF and efficiencies for the DSSCs employing various dyes and mediators.

**Table 2:** Efficiencies for different dyes and electrolytes

Dye	Redox couple (RC)—(a)/HTM—(b)	FF (%)	*η* (%)	Reference
LEG4 + ADEKA-1	^(a)^ Co^3+/2+^	77	14.3	[15]
D358	Tetra-n-propyl ammonium iodide	60	2.37	[311]
N719	^(a)^ I_3_^−^/I^−^	71	8.35	[362]
Y123	^(a)^ Co^3+/2+^	74	8.81	[101]
EosinY	^(a)^ Co(bpy)_3_	72	3.85	[363]
YD2-o-C8	^(a)^ Co^3+/2+^	68	8.97	[364]
Kojic acid-Azo 4	^(a)^ I_3_^−^/I^−^	75	1.54	[365]
N719	^(a)^ I_3_^−^/I^−^	72	8.57	[366]
N719	^(a)^ Co^3+/2+^	71	10.42	[367]
N719	^(a)^ I_3_^−^/I^−^	67	7.88	[368]
N719	^(a)^ I_3_^−^/I^−^	72	9.96	[369]
C106	^(a)^ T_2_^−^/T^−^	70	7.60	[370]
C106TBA	^(a)^ I_3_^−^/I^−^	74	9.54	[371]
YA422	^(a)^ Co^3+/2+^	74	10.65	[372]
SM315	^(a)^ Co^3+/2+^	78	13	[373]
Y123	^(a)^ Co^3+/2+^	71	10.30	[374]
Z907	^(a)^ T_2_^−^/T^−^	72	7.90	[246]
N3	^(a)^ I_3_^−^/I^−^	71	9.25	[375]
Y123	^(a)^ Co^3+/2+^	78	9.90	[376]
FNE29	^(a)^ Co^3+/2+^	70	8.24	[377]
CYC-B11	^(a)^ I_3_^−^/I^−^	67	9	[378]
2-TPA-R	^(a)^ I_3_^−^/I^−^	72	2.3	[184]
T1	^(a)^ I_3_^−^/I^−^	60	5.73	[379]
PTZ-1	^(a)^ I_3_^−^/I^−^	65.3	5.4	[150]
Y123 (OD)	^(b)^ Spiro-OMeTAD	76	7.2	[380]
N719	^(b)^ VM3	43	0.075	[381]
Z907	^(b)^ AS37	62	2.48	[382]
N3	^(b)^ Pentacene	49	0.8	[383]
D102 (OD)	^(b)^ 4d	32	0.54	[148]
SQ (OD)	^(b)^ TVT	64	0.19	[149]
D102 (OD)	^(b)^ VM5C9	38	0.47	[384]
N719	PET membrane	83	10.24	[138]
N719	LC-5% doped	61	4.61	[120]
Mangosteen	PEG: liquid electrolyte (Type I)	27	0.015	[106]
Mangosteen	PEG: liquid electrolyte (Type II)	14.5	0.010	[106]

### Developments in Dye Synthesis

As dyes play a key role in DSSCs, numerous inorganic and organic/metal-free dyes/natural dyes, like N3 [26], N719 [143], N749 (black dye) [144], K19 [145], CYC-B11 [146], C101 [32], K8 [147], D102 [148], SQ [149], Y123 [101], Z907 [150], Mangosteen [106], and many more have been utilized as sensitizers in DSSCs. Few of them will be discussed below briefly:

#### Metal (Ru) Complexes

Metal complex dyes produced from the heavy transition metals such as the complexes of ruthenium (Ru), Osmium (Os), and Iridium (Ir) have widely been used as inorganic dyes in DSSCs because of their long excited lifetime, highly efficient metal-to-ligand charge transfer spectra, and high redox properties. ML2(X)2 is the general structure of the sensitizer preferred as a dye, where M represents a metal, L is a ligand like 2,2′-bipyridyl-4,4′-dicarboxylic acid and X presents a halide, cyanide, thiocyanate, acetyl acetonate, and thiocarbamate or water substituent group [151]. Due to the thermal and chemical stability and wide absorption range from visible to NIR, the ruthenium polypyridyl complexes show best efficiencies and, thus, have been under extensive use so far.

##### Ru Complexes

**Fig. 12:**
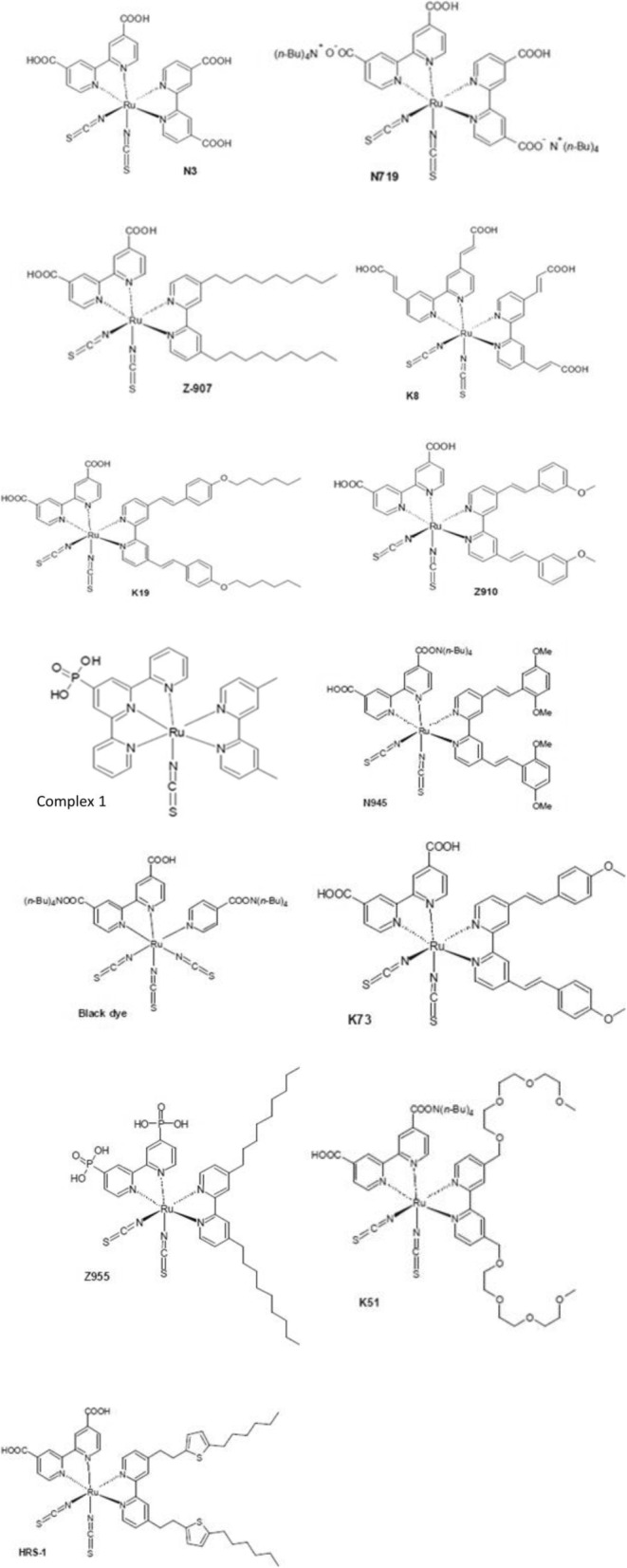
Molecular structure of Ruthenium complex based dye sensitizers

In 1991, O’Regan and Grätzel reported the efficiency of 7.12% for the very first DSSC based on the ruthenium dye (black dye) [3]. Later, an efficiency of about 10% was reported by them using Ru-based dye (N749) which has given this topic a new sight. Most of the Ru complexes consist of Ru(II) atoms coordinated by polypyridyl ligands and thiocyanate moieties in octahedral geometery, and because of the metal to ligand charge transfer (MLCT) transitions, they exhibit moderate absorption coefficient, i.e., < 18,000 M^− 1^ cm^− 1^. Ru (II) complexes lead the inter crossing of excited electron to the long lived triplet state and augmentation in the electron injection. Further, to improve the absorption and emission as well as electrochemical properties of Ru complexes, bipyridyl moieties can be replaced by the carboxylate polypyridine Ru dyes, phosphate Ru dyes, and poly nuclear bipyridyl Ru dyes. Table 3 and Fig. 12 show the molecular structure, the absorption spectra, and photoelectric performance for DSSCs based on different metal complex [polypyridyl (RuII)] dyes.

**Table 3:** Absorption spectra and photoelectric performance for DSSCs based on different metal complex [polypyridyl (RuII)] dyes

Dye	Absorption coefficient *ε* (10^3^m^2^mol^−1^)	IPCE (%)	*J*_SC_ (mAcm^−2^)	*V*_OC_ (*V*)	FF	*η* (%)	Ref.
N3	534	83	18.20	720	0.730	10.00	[385]
N719	532	85	17.73	846	0.750	11.18	[386]
N749 black dye	605	80	20.53	720	0.704	10.40	[387]
N749	–	80	20.90	736	0.722	11.10	[388],[389]
Z907	526	72	13.60	721	0.692	6.80	[390],[391]
Z907	526	72	14.60	722	0.693	7.30	[391]
K8	555	77	18.00	640	0.750	8.64	[392]
K19	543	70	14.61	711	0.671	7.00	[393]
N945	550	80	16.50	790	0.720	9.60	[394]
Z910	543	80	17.20	777	0.764	10.20	[168]
K73	545	80	17.22	748	0.694	9.00	[395]
K51	530	70	15.40	738	0.685	7.80	[396]
HRS-1	542	80	20.00	680	0.690	9.50	[397]
Z955	519	80	16.37	707	0.693	8.00	[398]

##### N3/N719/N712 Dyes

In 1993, Nazeeruddin et al. reported DSSC based on Ru-complex dye known as N3 dye {cis-di(thiocyanato)bis(2,2-bipyridine-4,4-dicarboxylate)ruthenium}, which contained one Ru center and two thiocyanate ligands (LL’) with additional carboxylate groups as anchoring sites and absorbed up to 800 nm radiations [26]. They obtained 10.3% efficiency for a system containing N3 dye and treated the dye covered film with TBP. At 518 and 380 nm wavelength, this dye attained maximum absorption spectra with respective extinction coefficients as 1.3 × 10^4^ M^− 1^ cm^− 1^ and 1.33 × 10^4^ M^− 1^ cm^− 1^, respectively. The dye has showed the 60 ns of excited state lifetime and sustained for more than 10^7^ turnovers without the significant decomposition since the beginning of the illumination [26]. Further, the absorption of the dye can be extended into the red and NIR by substituting the ligands such as thiocyanate ligands and halogen ligands. For example, a device containing acetylacetonate showed *η* = 6.0% [152], followed by a pteridinedione complex with 3.8% efficiency [153] and a diimine dithiolate complex with 3.7% efficiency [154].

It has been investigated that during esterification, the dye gets bounded to the TiO_2_ chemically which results in the partial transformation of protons of the anchoring group to the surface of the TiO_2_. Thus, it was concluded that the photovoltaic (PV) performance of the cell gets influenced by the presence of the number of protons on the N3 photosensitizer or, in other words, the modification in protonation level of N3 (N712, N719) affects the performance of the device [155, 156], in two major aspects. Firstly, the increase in the concentration of the protons results in the positively charged TiO_2_ surface and the downward shift in the Fermi level of TiO_2_. Hence, a drop in the *V*_OC_ takes place due to the positive shift of the conduction band edge induced by the surface protonation. Secondly, the electric field associated with the surface dipole enhances the absorption of the anionic Ru(II) complexes and, thus, insists the electron injection from the excited state of the dye to the conduction band of the TiO_2_. In 2001, Nazeeruddin et al. reported a 10.4% of efficiency for the DSSCs using a ruthenium dye, i.e., “black dye” [157], where its wide absorption band covers the entire visible range of wavelengths. Grätzel and group demonstrated the PCE of 9.3% for the monoprotonated sensitizer N3 [TBA]_3_ closely followed by a diprotonated sensitizer N3[TBA]_2_ or N719 with a conversion efficiency of 8.4% [156]. Later, Wang et al. and Chiba et al. reported a *η* = 10.5% [158] and *η* = 11.1% [159], for the devices that used black dye as a sensitizer in DSSCs.

A new dye “N719” was reported by Nazeeruddin et al. by replacing four H^+^ counterions of N3 dye by three TBA^+^ and one H^+^ counterions and achieved *η* = 11.2% for the respective device [155]. Despite having almost the same structure to the N3 dye, the higher value of *η* for N719 was accredited to the change in the counterions, as they altered the speed of adsorption onto the porous TiO_2_ electrode, i.e., N3 is fast (3 h) whereas N719 is slow (24 h). The dye-sensitized solar cell database (DSSCDB) yields around 329 results assembled from over 250 articles when queried as “N719,” where the reported efficiencies range between 2 and 11% [160].

**Fig. 13:**
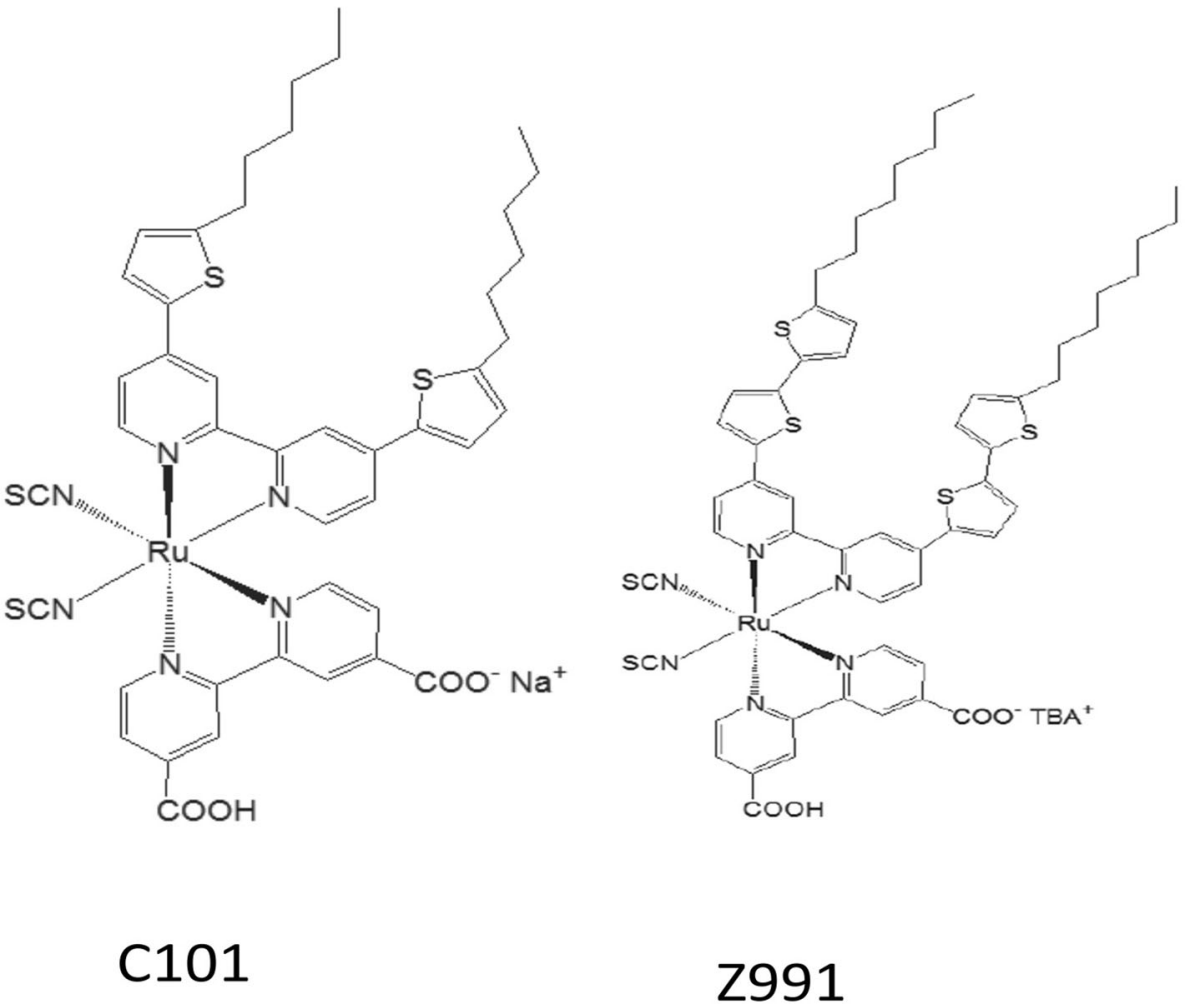
Molecular structure of C101 and Z991 sensitizers

**Fig. 14:**
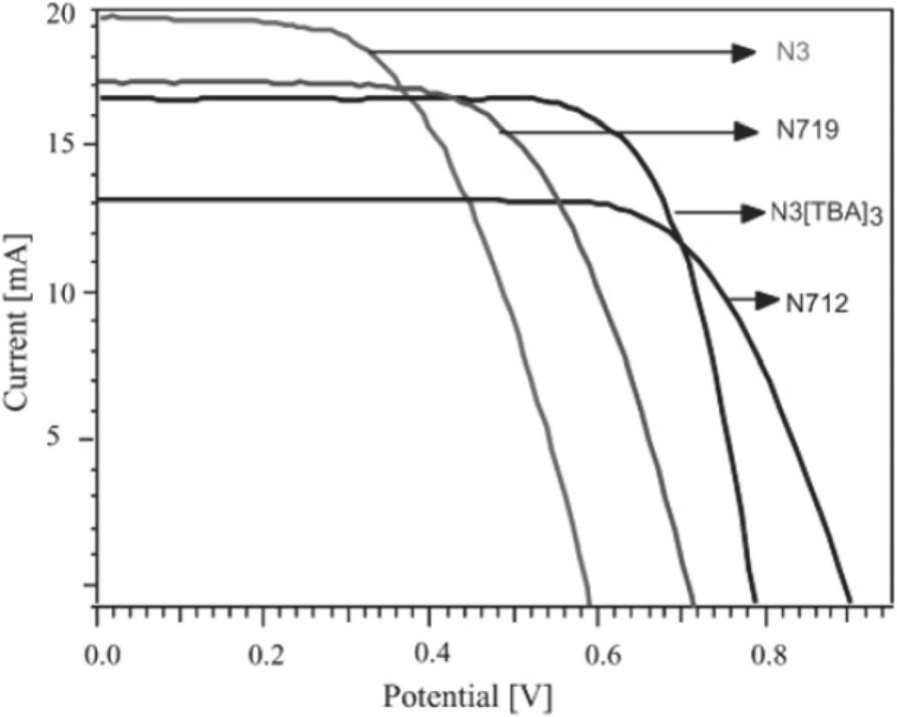
Effect of dye protonation on photocurrent-voltage characteristics of nanocrystalline TiO_2_ cell sensitized with N3 (4 protons), N719 (2 protons), N3[TBA]_3_ (1 proton), and N712 (0 protons) dyes, measured under AM 1.5 sun using 1 cm^2^ TiO_2_ electrodes with an I^−^/I_3_^−^ redox couple in methoxyacetonitrile [156]

Recently, Shazly et al. fabricated the solid-state dye-sensitized solar cells based on Zn_1-x_Sn_x_O nanocomposite photoanodes sensitized with N719 and insinuated with spiro-OMeTAD as a solid hole transport layer [161] and achieved highest efficiency of 4.3% with *J*_SC_ = 12.45 mAcm^− 2^, *V*_OC_ = 0.740 V, and FF = 46.70. Similarly, by applying different techniques, like post treatment of photoanode, optimizing the thickness of the nc-TiO_2_ layer, and the antireflective filming, Grätzel group reported *η* = 11.3% [32] for the device containing the dyes C101 and *η* = 12.3% [162] for Z991 dye-based DSSCs (molecular structure shown in Fig. 13). Again, if a sensitizer does not carry even a single proton, the value expected for *V*_OC_ will be high but the value for *J*_SC_ becomes low. Thus, there should be an optimal amount of protonation of the sensitizer required, so that the product of both *J*_SC_ and *V*_OC_ can determine the conversion efficiency of the cell as a maximum. And thus, deprotonation levels of N3, N719, and N712 in solar cells were investigated, where the doubly protonated salt form of N3 or N719 showed higher PCE as compared to the other two sensitizers [143]. Figure 14 shows the effect of dye protonation on the *I–V* characteristics of TiO_2_ photoanode sensitized with different Ru dyes as N3 (4 protons), N719 (2 protons), N3[TBA]_3_ (1 proton), and N712 (0 protons) dyes, measured under AM 1.5 [156]. However, the main limitations of N3 sensitizers are their relatively low molar extinction coefficient and less of absorption in the red region of the visible spectrum.

##### π-System Extension (N945, Z910, K19, K73, K8, K9)

As compared to the other organic dyes, standard Ru complexes have significantly lower absorption coefficient and thus a thick layer of TiO_2_ was required, which results in the higher electron recombination probability. Thus, two carboxylic acid groups of N3 can be replaced by the ligands containing conjugated π-systems to enhance the absorption and the cell efficiency, simultaneously. Thus, the reason behind the π-system extension in dyes is to create sensitizers with higher molar extinction coefficients (*ε*), so that the LUMO of the dye can be tuned to get directionality in the excited state and to introduce hydrophobic side chains that repel water and triiodide from the TiO_2_ surface. Recently, Rawashdeh et al. have demonstrated an efficiency of 0.45% by modifying the photoanode as graphene-based transparent electrode sensitized with 0.2 mM N749 dye in ethanolic solution [163].

Styryl-ligands attached to the bipyridil ring showed the utmost results. The *ε* = 1.69 × 10^4^ M^− 1^ cm^− 1^ for the Z910 dye [164], *ε* = 1.82 × 10^4^ M^− 1^ cm^− 1^ for the K19 dye [145], and *ε* = 1.89 × 10^4^ M^− 1^ cm^− 1^ for the N945 dye [165] have been found, which were at least 16% more as compared to the standard N3 dye. An efficiency of 10.2% was demonstrated by Wang et al. for Z910 dye [166]. 10.8% of the efficiency was observed for the N945 dye [167] in 2007, on thick electrodes and with volatile electrolytes which was about the same as for the N3 as reference, but, when applied on thin electrodes and with non-volatile electrolytes, the observed PCE was significantly higher. At the same time, a remarkable stability at 80 °C (in darkness) and 60 °C temperature (under AM 1.5) was observed [168], and between − 0.71 V and − 0.79 V vs. normal hydrogen electrode (NHE) [145, 165, 166, 168], the excited state of these dyes has been reported and was observed sufficiently more negative than the conduction band of TiO_2_ (ca. − 0.1 V vs. NHE) to ensure the complete charge injection. In terms of higher molar extinction coefficient, Nazeeruddin et al. synthesized K8 and K9 dyes that showed even better results as compared to the previous ones. K8 and K9 complexes showed broad and intense absorption bands between 370 and 570 nm. In DMF solution, the K9 complex showed the maxima at 534 nm (*λ*_max_) with a *ε* = 14,500 M^− 1^ cm^− 1^ which was blue shifted by 22 nm compared to K8 complex which showed maxima at 556 nm with a *ε* = 17,400 M^− 1^ cm^− 1^, respectively. Thus, due to the substitution of 4, 4′-bis (carboxylvinyl)-2, 2′-bipyridine by 4,4′- dinonyl-2,2′-bipyridine, *ε* observed for K9 complex was ~ 20% less than that of the K8 complex. The overall PCE observed for K8 and K9 complexes were 8.46% with *J*_SC_ = 18 mA/cm^2^ and *V*_OC_ = 640 mV and 7.81% with *J*_SC_ = 16 mA/cm^2^ and *V*_OC_ = 666 mV [169], respectively. Grätzel group synthesized K19 as a second amphiphilic dye and demonstrated that K19 shows 18,200 M^− 1^ cm^− 1^ M extinction coefficient, 7.0% overall conversion efficiency and a low energy metal-to-ligand transition (MLCT) absorption band at 543 nm, which was higher than the corresponding values for the first amphiphilic dye Z907 with a molar extinction coefficient of 12,200 M^− 1^ cm^− 1^, 6.0% overall conversion efficiency, and standard N719 dye with a molar extinction coefficient of 14,000 M^− 1^ cm^− 1^ with 6.7% overall PCE under the same fabrication and evaluation conditions. They appraised the performance of the device using N719, Z907, and K19 as sensitizers during thermal aging at 80 °C and observed a lower stability for N719 dye may be due to the desorption of the sensitizer at higher temperature; however, K19 and Z907, both retained over 92% of their initial performances under the thermal stress at 80 °C for 1000 h [145, 169].

Thiophene ligands containing Ru sensitizers also showed good efficiencies. In 2006, Yanagida et al. reported a Ru complex, by replacing a phenylvinyl group of K19 by thienylvinyl group in HRS-1 [170] and an improved stability along with respectable LHE in vis-NIR and a reversible one electron oxidation process was reported. They found a *η* up to 9.5% for HRS-1 (substituted thiophene derivatives). Several thiophene containing sensitizers have been developed without conjugation, such as C101 [32] and CYC-B1 [171]. After the development of C101 dye, Ru (II) thiophene compounds gained special attention as having set a new DSSC efficiency record of 11.3–11.5% and became the first sensitizer to triumph over the well-known N3 dye [32].

##### Amphiphilic Dyes with Alkyl Chains

Two of the four carboxylic groups of N3 dye are replaced by long alkyl chains because the ester linkage of the dye to the TiO_2_ was prone to hydrolyze, if water gets adsorbed on the TiO_2_ surface [172], thus resulting in usually lower absorption spectrum in these sensitizers due to the smaller conjugated π-system of the bipyridil-ligand. Even though the PCE offered by these sensitizers were appreciable, ranging from 7.3% for Z907 (with 9 carbon atoms) [54] to 9.6% for N621 (with 13 carbon atoms) [155] and were highly stable, Z907 sensitized DSSCs passed 1000 h at 80 °C in darkness and at 55 °C under illumination without any degradation [173]. It has been found that by coadsorption of decylphosphonic acid on the TiO_2_ NPs, the hydrophobicity of the surface could be even enhanced and, thus, stable cells have been demonstrated [54].

##### Different Anchoring Groups

Most of the sensitizers in DSSCs have carboxylic acid groups as an anchor on the surface of TiO_2_. But, the dye molecules get desorbed at the semiconductor surface at a pH > 9, due to the shifting of the equilibrium towards the reactant side. Thus, dyes with different anchoring groups are much needed. Again, most of the research focuses on phosphonic acid and the credit goes to its binding strength to a metal oxide surface, as the binding strength to a metal oxide surface decreases in the order, phosphonic acid > carboxylic acid > ester > acid chloride > carboxylate salts > amides [174]. Z955 is a Ru-complex containing phosphonic acid as an anchoring group and demonstrated a *η* = 8% accompanied by good stability under prolonged light soaking for about 1000 h at AM 1.5 and 55 °C [175]. Triethoxysilane [176] and boronic acid are some other anchoring groups. π-Extended ferrocene with varied anchoring groups (–COOH, –OH, and –CHO) has been applied as photosensitizers in DSSCs [177]. Chauhan and co-workers has synthesized and characterized two new compounds as FcCH=NC_6_H_4_COOH (1) and FcCH=NCH_2_CH_2_OH (2), where Fc = C_5_H_4_FeC_5_H_5_ and FcCHO are used as the starting material [177]. By cyclic voltammetry (CV) in dichloromethane solution and using density functional theory (DFT) calculations, they have explained the quasi-reversible redox behavior of the dyes. The redox-active ferrocenyl group exhibited a single quasi-reversible oxidation wave with E′ = 0.34, 0.44, and 0.44 V for 1, 2, and 3, respectively. In 2017, a study was carried out to inspect the influence of a cyano group in the anchoring part of the dye on its adsorption stability and the overall PV properties like electron injection ability to the surface and *V*_OC_ [178]. The results indicated that the addition of the cyano group increased the stability of adsorption only when it adsorbs via CN with the surface and it decreased the photovoltaic properties when it was not involved in binding.

**Fig. 15:**
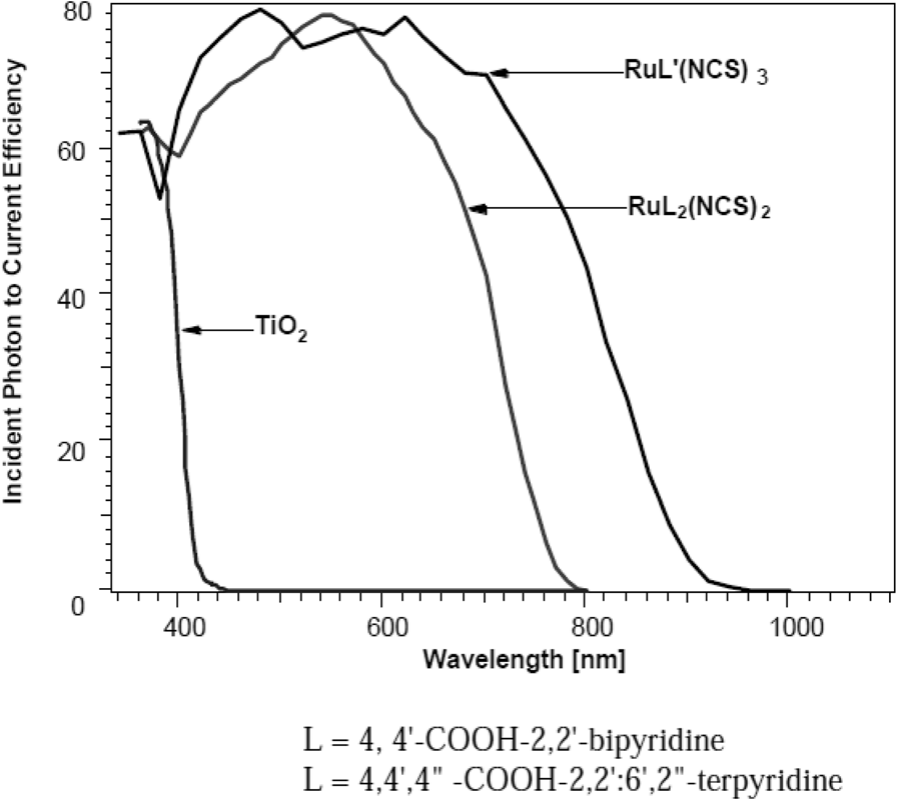
Photocurrent action spectra obtained with the N3 (ligand L) and the black dye (ligand L_) as sensitizer. The photocurrent response of bare TiO^2^ films is also shown for comparison [26]

However, in the race of improved efficiency and efficient DSSCs, Ru (II) dyes are still an ace. The most vital reason following usage of Ru dyes in DSSCs is their extraordinary stability when being absorbed on the TiO_2_ surface. N749 and Z907 are the two important Ru dyes, although N749 which shows broad absorption and high efficiency, in contrast, has low absorption coefficient about ~ 7000 M^− 1^ cm^− 1^ and the stability of this dye was not so good as compared to other Ru sensitizers. PCE of 10.4% has been observed for black dye, under AM 1.5 and full sunlight [48]. It achieved sensitization over the whole visible range extending into the NIR up to 920 nm with 80% IPCE and 10.4% overall efficiency, when anchored on TiO_2_ nanocrystalline film. At NREL, black dye (N749)-sensitized DSSC showed efficiency of 10.4% with *J*_SC_ = 20.53 mA/cm^2^, *V*_OC_ = 0.721 V, and FF = 0.704, where the active area of the cell was 0.186 cm^2^ [157, 179]. Nazeeruddin et al. reported a comparative study between the spectral response of the photocurrent of the two dyes, N3 and N749 [26], as shown in Fig. 15. In the vis-range, both chromophores showed very high IPCE values. The response of N749 dye was observed to be extended 100 nm further into the IR compared to that of N3. The recorded photocurrent onset was close to 920 nm and there on the IPCE rose gradually until at 700 nm it reached to a plateau of ca. 80%. From overlap integral of the curves in Fig. 8 with the AM 1.5 solar emission, it could be predicted that *J*_SC_ of the N3 and black dye-sensitized cells to be 16 and 20.5 mA/cm^2^, respectively [37]. In Z907 sensitizer, one of the dicarboxy bipyridine ligands in N3 molecule was replaced by a nonyl bipyridine, which resulted in the formation of a hydrophobic environment on the device. However, the dye has set a precedent for hundreds of tris-heteroleptic Ru complexes with isothiocyanate ligands that were developed in the last 15 years, but provides efficiencies rarely comparable to N719 [180].

#### Metal-Free, Organic Dyes

**Fig. 16:**
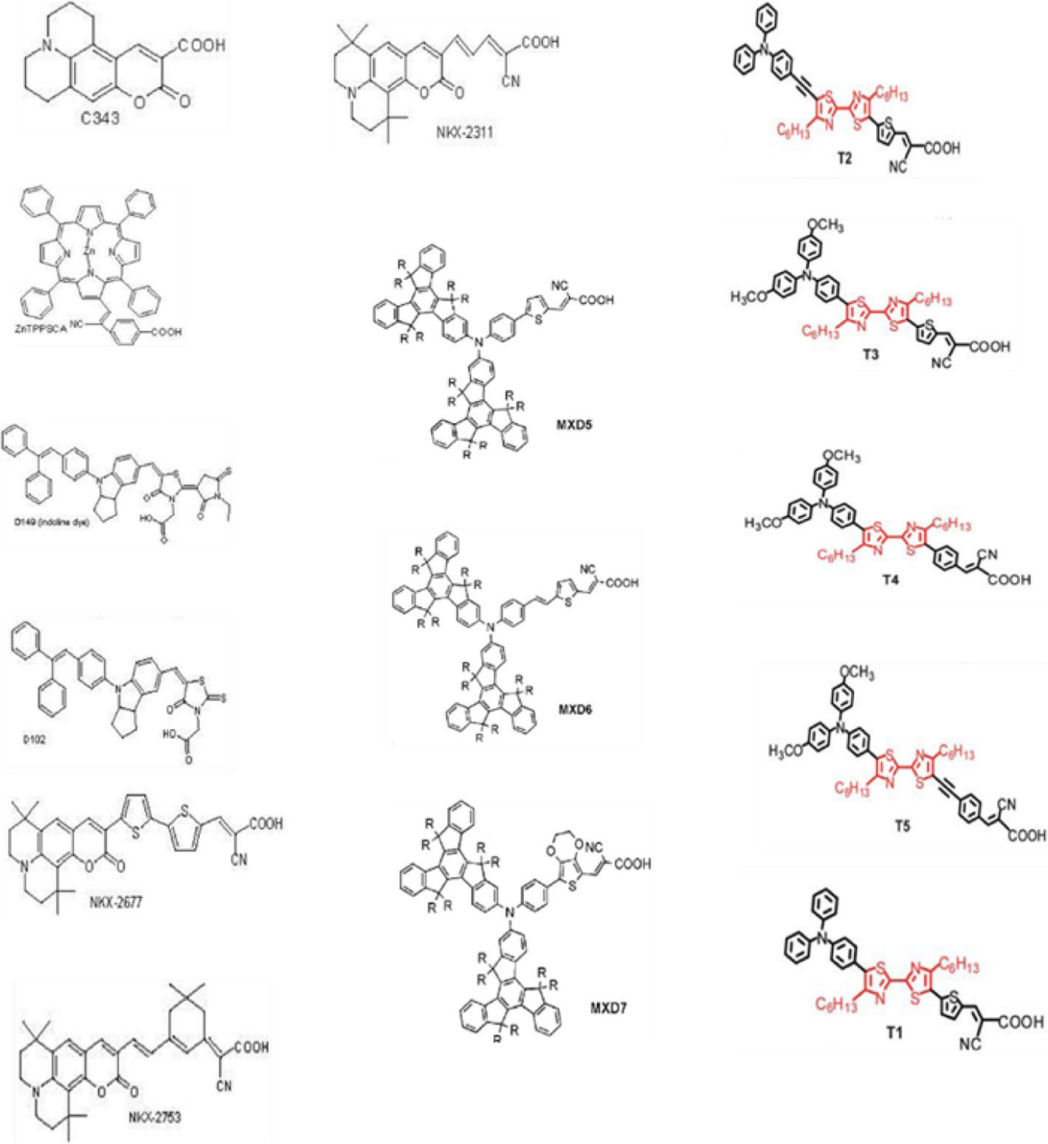
Molecular Structure of metal-free organic dyes

Despite the capability to provide highly efficient DSSCs, the range of application of Ru dyes are limited to DSSCs, as Ru is a rare and expensive metal and, thus, not suitable for cost-effective, environmentally friendly PV systems. Therefore, development and application of new metal-free/organic dyes and natural dyes is much needed. The efficiency of DSSCs with organic dyes has been increased significantly in the last few years and an efficiency of 9% [181] was shown by Ito and co-workers. The molecular structure and the efficiency for DSSCs based on different metal-free organic dyes are shown in Table 4 and Fig. 16.

**Table 4:** The efficiency for DSSCs based on different metal-free organic dyes

Dye	Derivative	Efficiency (%)	Reference
NKX-2311	Coumarin	5.6	[198]
NKX-2753	Coumarin	6.7	[399]
NKX-2677	Coumarin	7.4	[222]
D5	TPA	5.1	[241]
D149	Indole	8	[200]
DPI-T	BPI	1.28	[400]
D149	Indole	9.0	[181]
ZnTPPSCA	Porphyrin	4.2	[401]
Zn2	Porphyrin	4.8	[402]
Zn3	Porphyrin	5.6	[232]
2TPA-R	TPA	2.3	[184]
RD-Cou	Coumarin	4.24	[225]
IK3	Indole	6.3	[230]
LD4	Zinc porphyrins	10.06	[235]
L2	TPA	3.08	[218]
MXD 7	TPA	6.18	[243]
Y123	TPA	10.3	[246]
T2-1	PTZ	5.5	[251]
PTZ-I	PTZ	5.4	[150]
S2	Carbazole	6.02	[185]
DPP-I	DPP core	4.14	[262]

Thus, metal-free organic dyes are developing at a fast pace to overcome the limitations discussed above and especially promising is the fast learning curve, which raises hope of the further synthesis of new materials with higher stability and, thus, designing highly efficient DSSCs at much lower prices. Though the efficiencies offered by these dyes are less comparable to those by Ru dyes, their application is vast as they are potentially very cheap because of the incorporation of rare noble metals in organic dyes; thus, their cost mainly depends on the number of synthesis steps involved. Other advantages associated with organic dyes are their structure variations, low cost, simple preparation process, and environmental friendliness as compared to Ru complexes; also, the absorption coefficient of these organic dyes is typically one order of magnitude higher than Ru complexes which makes the thin TiO_2_ layer feasible. Thus, there is a huge demand to develop new pure organic dyes, so that the commercialization of DSSCs becomes easier.

**Fig. 17:**
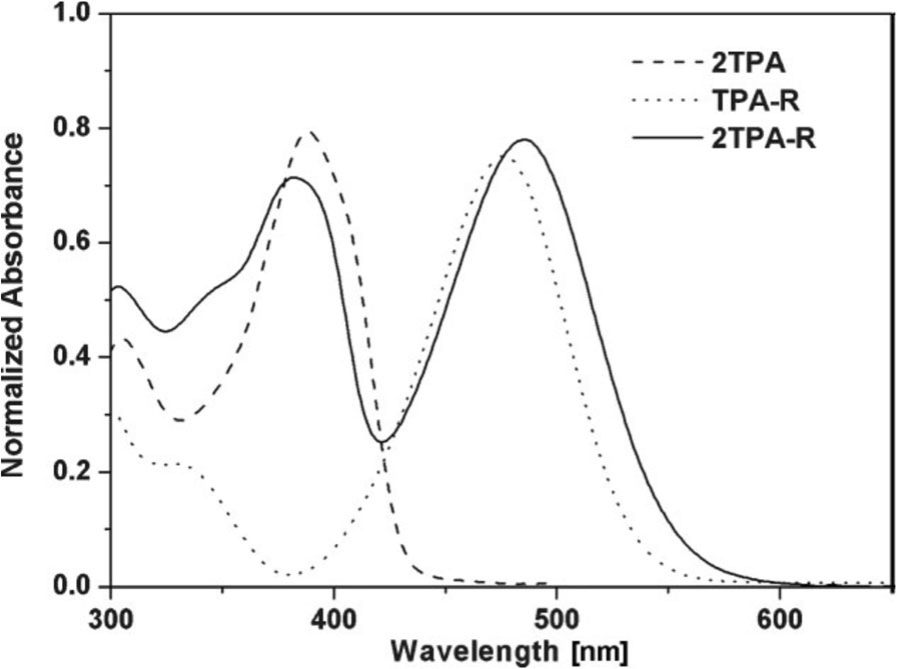
Absorption spectra of 2TPA, TPA-R, and 2TPA-R in CH_2_Cl_2_ solutions [184]

However, there are certain limitations associated with these dyes too, as under high elevated temperatures the observed stability of the organic dyes were not as good as expected. Therefore, to get a larger photocurrent response for newly designed and developed organic dyes, it is essential to attain predominant light-harvesting abilities in the whole visible region and NIR of dyes, with a sufficiently positive HOMO than I^−^/I^−^ _3_ redox potential and sufficiently negative LUMO than the conduction band edge level of the TiO_2_ semiconductor, respectively [182, 183]. In 2008, Tian et al. fabricated DSSCs based on a novel dye (2TPA-R), containing two triphenylamine (TPA) units connected by a vinyl group and rhodanine-3-acetic acid as the electron acceptor to study the intramolecular energy transfer (E_n_T) and charge transfer (ICT) [184]. They found that the intramolecular E_n_T and ICT processes showed a positive effect on the performance of DSSCs, but the less amount of dye was adsorbed on TiO_2_ which may make it difficult to improve the efficiency of DSSCs [184]. An efficiency of 2.3% was attained for the DSSC used 2TPA-R dye and an imidazolium iodide electrolyte, whereas *η* = 2% was achieved for TPA-R dye. This improved efficiency for 2TPA-R device was possibly due to the contribution of the E_n_T and ICT. They studied the effect of 2TPA-R via absorption spectrum (as shown in Fig. 17) and found that the two absorption bands, i.e., *λ*_abs_ at 383 nm and 485 nm obtained for 2TPA-R, are almost the same as those observed for 2TPA (*λ*_abs_ at 388 nm) and TPA-R (*λ*_abs_ at 476 nm) in CH_2_Cl_2_ solution (2 × 10^− 5^ M). Thus, the study on intramolecular E_n_T and ICT could help in the design and synthesis of efficient organic dyes. Hence, a suitable anchoring group which can chemically bind over the TiO_2_ surface with a suitable structure and effective intramolecular E_n_T and ICT processes, is also required for synthesis.

The construction of most of the organic dyes is based on the donor- acceptor (D-A)-like structure linked through a π-conjugated bridge (D–π–A) and usually has a rod-like configuration. Moieties like indoline, triarylamine, coumarin, and fluorine are employed as an electron donor unit, whereas carboxylic acid, cyanoacrylic acid, and rhodamine units are best applicable as electron acceptors to fulfill the requirement. The linking of donor and acceptor is brought about by adding π spacer such as polyene and oligothiophene [185, 186]. This type of the structure results in a higher photoinduced electron transfer from the donor to acceptor through linker (spacer) to the conduction band of the TiO_2_ layer, where the π-conjugation can be extended either by increasing the methine unit or by introducing aromatic rings such as benzene, thiophene, and furan or in other words by adding either electron donating or withdrawing groups, which results in the enhanced light harvesting ability of the dye, and by using different donor, linker, and acceptor groups, the photophysical properties of the organic dyes can also be tuned [187, 188]. Whereas the photophysical properties change with the expansion of π-conjugation due to the shift of the both HOMO and LUMO energy levels, thus, D–π–A structure was considered to be the most promising class of organic dyes in DSSCs as they can be easily tuned [189]. Moreover, in 2010, the encouraging efficiency up to 10.3% was reported using organic dyes [190]. Fuse et al. demonstrated a one-pot procedure to clarify the structure–property relationships of donor–π–acceptor dyes for DSSCs through rapid library synthesis [191]. Four novel organic dyes IDB-1, ISB-1, IDB-2, and ISB-2, based on 5-phenyl-iminodibenzyl (IDB) and 5-phenyliminostlbene (ISB) as electron donors and cyanoacrylic acid moiety as an electron acceptor connected with a thiophene as a π-conjugated system, were designed by Wang et al. in 2012 [192]. The highest efficiencies for the devices based on ISB-2 were observed due to the larger red shifts of 48 nm for ISB-2, indicating the more powerful electron-donating ability due to the increased linker conjugation. The absorption peaks for IDB-1, ISB-1, IDB-2, and ISB-2 were obtained at 422, 470, 467, and 498 nm in dichloromethane-diluted solution, respectively.

Tetrahydroquinolines [193, 194], pyrolidine [195], diphenylamine [196], triphenylamine (TPA) [27, 197], coumarin [198, 199], indoline [200, 201], fluorine [202], carbazole (CBZ) [203], phenothiazine (PTZ) [204, 205], phenoxazine (POZ) [206], hemicyanine dyes [207], merocyanine dyes [208], squaraine dyes [209], perylene dyes [210], anthraquinone dyes [211], boradiazaindacene (BODIPY) dyes [212], oligothiophene dyes [213], and polymeric dyes [214] are widely used in DSSCs and are still under development. Jia et al. designed quasi-solid-state DSSCs employing two efficient sensitizers FNE55 and FNE56, based on fluorinated quinoxaline moiety, i.e., 6, 7-difluoroquinoxaline moiety, and an organic dye FNE54 without fluorine was designed for comparison [215]. From the studies, it was concluded that the absorption properties of the dye enhanced bathochromically from 504 nm for FNE54 to 511 nm and 525 nm for FNE55 and FNE56 sensitizers upon the addition of fluorine into the dye. The addition of fluorine resulted in the improved electron-withdrawing ability of the quinoxaline and, thus, enhanced the push–pull interactions and narrowed the energy band gap. Due to the high polarizability, spectroscopically and electrochemically tunable properties and high chemical stability, π-conjugated oligothiophenes were well applied as spacers in DSSCs [194, 204]. To induce a bathochromic shift and augment the absorption, a number of thiophene units could be increased in the spacers, and by controlling the length of these thiophene units or chain, higher efficiencies up to two to three units can be achieved [216, 217], as the π-conjugated spacers used previously were thiophenes linked directly or through double bonds to the donor moiety [218].

As good electron injection is one of the parameters for higher efficiency in the DSSCs, cyanoacetic acid and cyanoacrylic acid are well employed as acceptor units due to their strong electron withdrawing capability. Yu et al. concluded cyanoacrylic acid as a strong electron acceptor for D–π–A-based dyes because the dye incorporating cyanoacrylic acid as an electron acceptor showed the best results and, due to the maximum absorption spectrum and the highest molar excitation coefficient, the DSSC achieved *η* = 4.93% [219]. Wang and co-workers designed organic dyes based on thienothiophene as π conjugation unit, where they used triphenylamine as donor and cyanoacetic acid as an acceptor. They substituted different alkyl chains on the triphenylamine unit and found the best efficiency of about 7.05% for the sensitizers with longer alkoxy chains due to the longer electron lifetime [220]. Acceptors based on rhodanine-3-acetic acid were also used as an alternative, but due to the low lying molecular LUMO, the results obtained were not pleasing [221, 222].

##### Coumarin Dyes

**Fig. 18:**
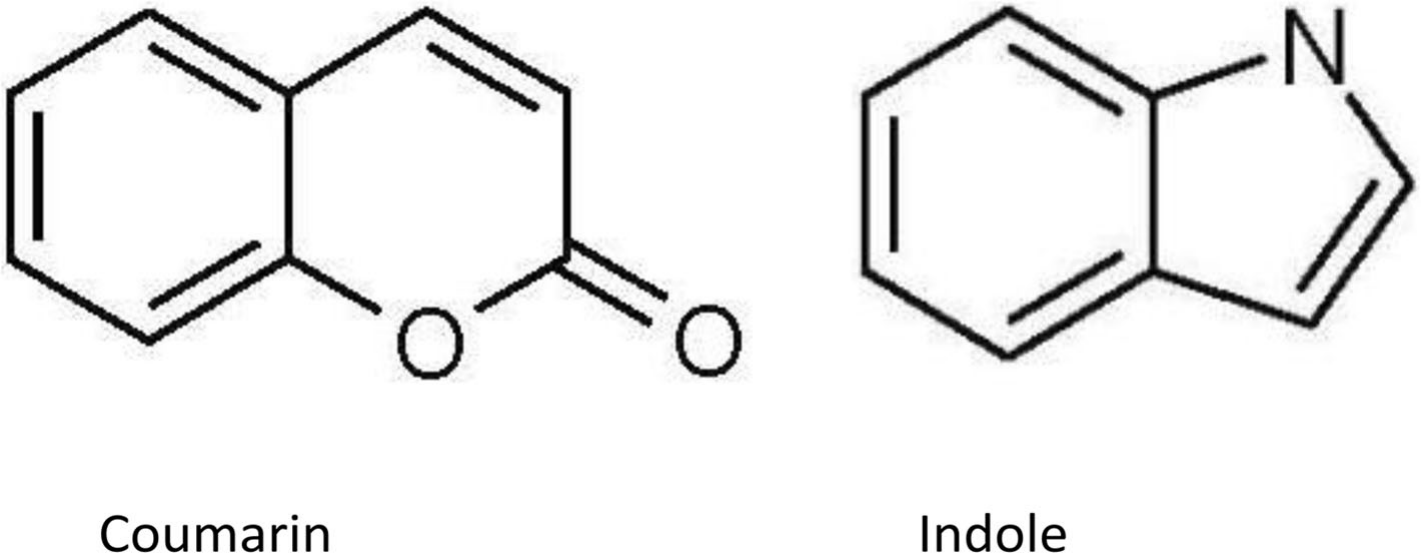
Molecular structure of **a** Coumarin and **b** indole

Coumarin is a synthetic organic dye and is a natural compound found in many plants like tonka bean, woodruff, and bison grass (molecular structure shown in Fig. 18a). In 1996, Grätzel et al. found the efficient electron injection rates of 200 fs from C343 into the conduction band of the TiO_2_, where for the first time the transient studies on a coumarin dye in DSSCs were performed [223]. But the narrow absorption spectrum of C343, i.e., lack of absorption in the visible region, resulted in the lower conversion efficiency of the device. This can be altered by adding more methane groups that result in expanding the π-conjugation linkers and an increased efficiency of the DSSC [224]. Giribabu and co-workers synthesized RD-Cou sensitizers and obtained the conversion efficiency of 4.24% using liquid electrolyte, where coumarin moiety was bridged to the pyridyl groups by thiophene, which resulted in the extended π-conjugation and broadening of the metal-to-ligand charge transfer spectra [225]. They found that the absorption spectrum of RD-Cou dye was centered at 498 nm with a *ɛ* = 16,046 M^− 1^ cm^− 1^. Despite the lower efficiency offered by these cells, the thermal stability of the sensitizer make its rooftop applications possible because the dye showed stability of up to 220 °C during thermal analysis.

##### Indole Dyes

Indole occurs naturally as a building block in amino acid tryptophan, and in many alkaloids and dyes too (molecular structure shown in Fig. 18b). It is substituted with an electron withdrawing anchoring group on the benzene ring and an electron donating group on the nitrogen atom, and these dyes have demonstrated good potential as a sensitizer. Generally, the D–A structure of an indole dye is such that the indole moiety acts as an electron donor and is connected to a rhodanine group that acts as an electron acceptor. Also by introducing the aromatic units into the core of the indoline structure, the absorbance in the infrared (IR) region of the visible spectra as well as the absorption coefficient of the dye can be enhanced significantly [226]. An efficiency of 6.1% was demonstrated for DSSCs with D102 dye, and by optimizing the substituents, 8% of the efficiency was attained with D149 dye [200]. Another dye “D205” was synthesized by controlling the aggregation between the dye molecules, as an indoline dye with an n-octyl substituent on the rhodanine ring of D149 [227]. They investigated that n-octyl substitution increased the *V*_OC_ without acknowledging the presence of CDCA too much. However, the increase in the *V*_OC_ of D205 due to the CDCA was approximately 0.054 V but showed little effect on D149 with an increase of 0.006 V only. But the CDCA and n-octyl chain (D205) together improved the *V*_OC_ by up to 0.710 V significantly, which was 0.066 V higher (by 10.2%) than that of D149 with CDCA.

**Fig. 19:**
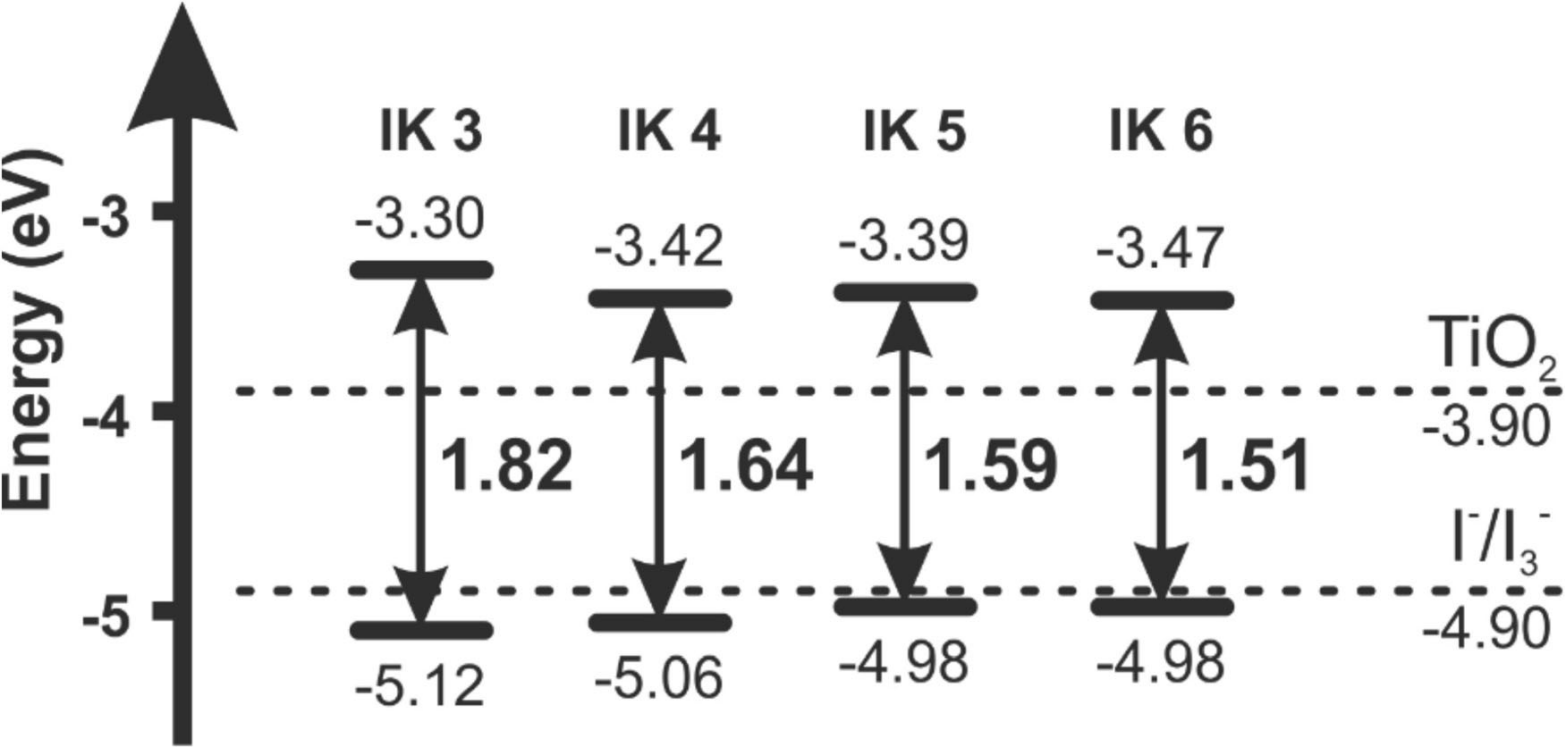
HOMO and LUMO energy level diagram of dyes IK 3–6 [230]

Further in 2012, *η* = 9.4% was shown by Wu et al. with the observed *J*_SC_ = 18 mAcm^− 2^, *V*_OC_ = 0.69 V, and FF = 0.78, by employing indoline as an organic dye in the respective DSSC [228]. Suzuka et al. fabricated a DSSC sensitized with indoline dyes in conjunction with the highly reactive but robust nitroxide radical molecules as redox mediator in a quasi-solid gel form of the electrolyte. They obtained an appreciable efficacy of 10.1% at 1 sun. To suppress a charge-recombination process at the dye interface, they introduced long alkyl chains, which specifically interact with the radical mediator [229]. Recently in 2017, Irgashev et al. synthesized a novel push-pull thieno[2,3-b]indole-based metal-free dyes and investigated their application in DSSCs [230]. They designed IK 3–6 dyes based on the thieno[2,3-b]indole ring system, bearing various aliphatic substituents such as the nitrogen atom as an electron-donating part, several thiophene units as a π-bridge linker, and 2-cyanoacrylic acid as the electron-accepting and anchoring group. An efficiency of 6.3% was achieved for the DSSCs employing 2-cyano-3-{5-[8-(2-ethylhexyl)-8H-thieno[2,3-b]indol-2-yl]thiophen-2-yl}acrylic acid (IK 3), under simulated AM 1.5 G irradiation (100 mWcm^− 2^), whereas the lower values of *η* = 1.3% and 1.4%, respectively, were shown by the dyes IK 5 and IK 6. The LUMO energy levels are more negative than the conduction edge of the TiO_2_ (− 3.9 eV), and their HOMO energy levels of all four dyes were found to be more positive than the I^−^/I_3_^−^ redox couple (− 4.9 eV), making possible regeneration of oxidized dye molecules after injection of excited electrons into TiO_2_ electrode (as shown in Fig. 19) [230]. The less efficiency of other dyes was contributed by the intermolecular π-stacking and aggregation processes in these dyes, proceeding on the photoanode surface.

##### Porphyrins

**Fig. 20:**
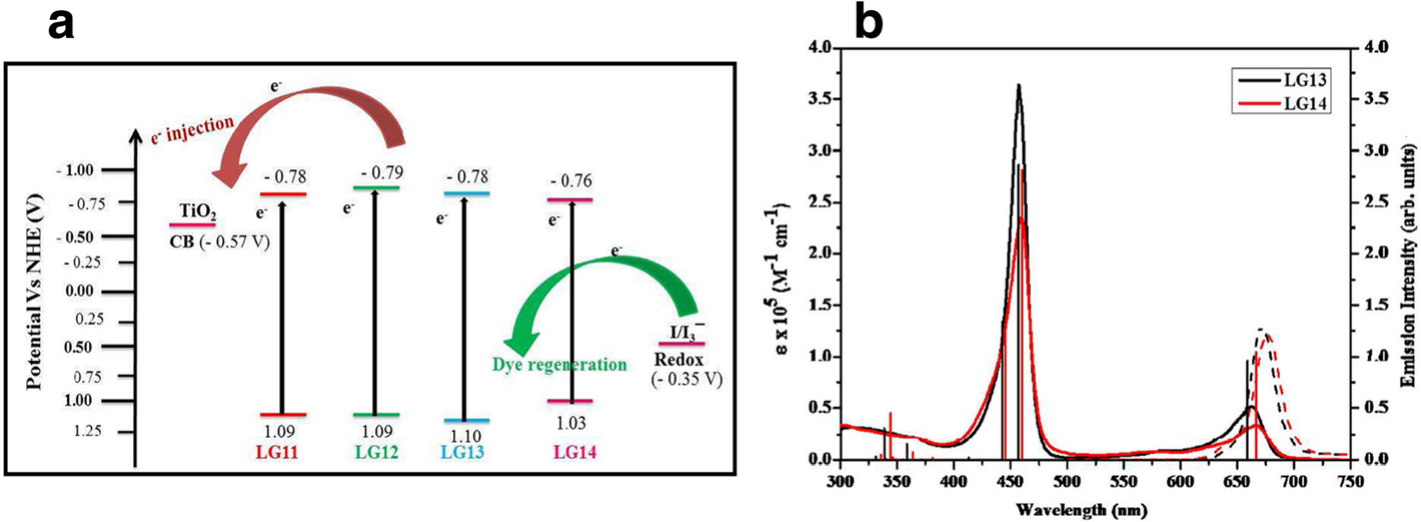
Energy level diagram of LG-11 to LG-14 porphyrins, electrolyte and TiO_2_ (**a**) and absorption (left, solid line) and emission (right, dashed line) spectra of porphyrin sensitizers LG-13 and LG-14 in the THF solvent (**b**)

Porphyrin shows strong absorption and emission in the visible region and has a long lifetime in its excited singlet state (> 1 ns), very fast electron injection rate (femtosecond range) [231], millisecond time scale electron recombination rate [232], and tunable redox potentials [13]. In 1987, the first paper was published on DSSCs based on efficient sensitization of TiO_2_ with porphyrins [233]. This led researchers in the direction to make efforts for the synthesis of novel porphyrin derivatives with the underlying idea to mimic nature’s photosystems I and II, so that the large molar extinction coefficient of the Soret bands and Q bands can be exploited. In 2007, a Zn-porphyrin dye-based DSSC was fabricated by Campbell et al. and has given the exceptional PCE of 7.1% [195]. Krishna and co-workers investigated the application of bulky nature phenanthroimidazole-based porphyrin sensitizers in DSSCs [234]. The group designed a novel D–π–A-based porphyrin sensitizer having strong electron-donating methyl phenanthroimidazole ring and ethynylcarboxyphenyl group at meso-position of porphyrin framework (LG11). They have attached the hexyl phenyl chains to the phenanthroimidazole moiety to reduce the unwanted loss of *V*_OC_ caused by dye aggregation and charge recombination effect, thus achieving an increase in *V*_OC_ to 460 and 650 mV. The energy level diagram and the absorption–emission spectra for the sensitizers (LG11-14) are shown in Fig. 20 [234].

**Fig. 21:**
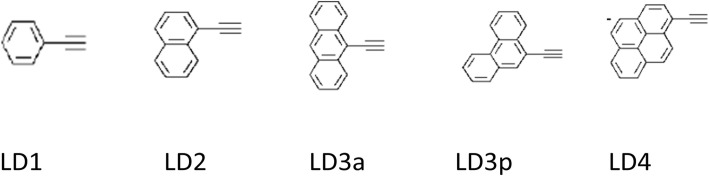
Molecular structures of LD porphyrins

Wang et al. have synthesized zinc porphyrins in a series bearing a phenylethynyl, naphthalenylethynyl, anthracenylethynyl, phenanthrenylethynyl, or pyrenylethynyl substituent, namely LD1, LD2, LD3a, LD3p, and LD4, as photosensitizers for DSSCs (as shown in Fig. 21). The overall efficiencies of the corresponding devices resulted as LD4 (with *η* = 10.06%) > LD3p > LD2 > LD3a > LD1. The higher value of *η* and *V*_OC_ = 0.711 V was achieved for LD4 due to the broader and more red-shifted spectral feature; thus, the IPCE spectrum was covered broadly over the entire visible region [235]. Later for a push–pull zinc porphyrin DSSCs, changes in the structural design were carried out and structures with long alkoxyl chains enveloping the porphyrin core were built. By following the process, a *η* = 12.3% was achieved by Yella et al. for DSSC with cobalt as the mediator [236].

**Fig. 22:**
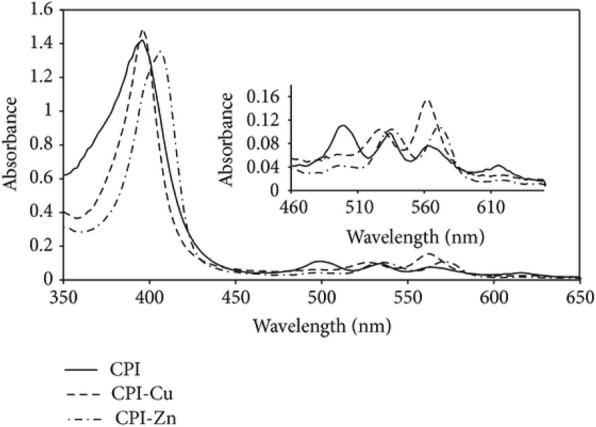
UV-vis spectra and in insert Q-band magnification for CPI, CPICu, and CPIZn incorporated into the TiO_2_ films [237]

Giovannetti et al. investigated the free base, Cu(II) and Zn(II) complexes of the 2,7,12,17-tetrapropionic acid of 3,8,13,18-tetramethyl-21H,23H porphyrin (CPI) in solution and bounded to transparent monolayer TiO_2_ nanoparticle films to determine their adsorption on the TiO_2_ surface, to measure the adsorption kinetics and isotherms, and to use the obtained results to optimize the preparation of DSSC PVCs (photovoltaic cells) [237]. The absorption spectra study of CPI, CPIZn, and CPICu molecules onto the TiO_2_ surface (as shown in Fig. 22) revealed the presence of typical strong Soret and weak Q bands of porphyrin molecules in the region 400–450 nm and 500–650 nm, which were not changed with respect to the solution spectra. They observed no modification in the structural properties of the adsorbed molecules.

##### Triarylamine Dyes

Due to the good electron as well as transporting capability and its special propeller starburst molecular structure with a nonplanar configuration, the triarylamine group is widely applied as a HTM in various electronic devices. Triarylamine derivative distributes the π–π stacking and, thus, improves the cells performance by reducing the charge recombination, minimizing the dye aggregation and enhancing the molar extinction coefficient of the organic dye [202, 217, 238]. By the addition of alkyl chains or donating groups, the structural modification of the triarylamine derivatives could be performed [218, 220, 239]. The performance of a basic D–π–A organic dye can be improved by simply binding donor substitutions on the π-linker of the dye [240]. Thus, Prachumrak and co-workers have synthesized three new molecularly engineered D–π–A dyes, namely T2–4, comprising TPA as a donor, terthiophene containing different numbers of TPA substitutions as a π-conjugated linker and cyanoacrylic acid as an acceptor [240]. To minimize the electron recombination between redox electrolyte and the TiO_2_ surface as well as an increase the electron correction efficiency, the introduction of electron donating TPA substitutes on the π-linker of the D–π–A dye can play a favorable game, leading to improved *V*_OC_ and *J*_SC_, respectively [240]. In 2006, Hagberg et al. published a paper on TPA-based D5 dye [241], where the overall PCE demonstrated for D5 dye was 5.1% in comparison with the standard N719 dye with an efficiency of 6.40% under the similar fabrication conditions. Thus, D5 appeared as an underpinning structure to design the next series of TPA derivatives.

In 2007, a series L0-L4 of TPA-based organic dyes were published by extending the conjugation in a systematic way [218]. By increasing the π conjugation, the absorption spectra and molar extinction coefficients of L0-L4 were increased. The observed IPCE spectra for L0 and L1 dyes were high, but the spectra of these dyes were not broad; as a result, lower conversion efficiencies were obtained for L0 and L1, whereas the broad absorption spectrum as well as the broad IPCE was obtained for L3 and L4 by the augmentation of linker conjugation, but the efficiencies observed were less than the L0 and L1 due to the amount of dye loading, i.e., with the increase in the size of dye there appears a decrease in the dye amount. Thus, the lower IPCE obtained for longer L3 and L4 may be accredited to unfavorable binding with the TiO_2_ surface. Higher efficiencies were obtained for solar cells based on L1 and L2, 2.75% and 3.08%, respectively. Baheti et al. synthesized DSSCs based on nanocrystalline anatase TiO_2_ and simple triarylamine-based dyes containing fluorene and biphenyl linkers [242]. They reported that the fluorene-based dyes showed better solar cell parameters than those of the biphenyl analogues. In 2011, Lu et al. reported the synthesis and photophysical/electrochemical properties of three functional triarylamine organic dyes (MXD5-7) as well as their application in dye-sensitized solar cells. They used the nonplanar structures of bishexapropyltruxeneamino as an electron donor [243] and investigated the impact of addition of chenodeoxycholic acid (CDCA) in the respective dyes, as MXD5-7 without CDCA showed lower photocurrent and efficiency as compared to the dyes MXD5-7 with 3 mM CDCA. However, the highest efficiency of 6.18% was observed for MXD7 (with 3 mM CDCA) with electron lifetime (*τ*) = 63 ms, under standard global AM 1.5 solar conditions (molecular structure is given in Table 4, where R = propyl).

Using furan as a linker, different TPA-based chromophores were studied by Lin and co-workers [244]. When D5 and its furan counterpart were compared, the results were exciting, still the light harvesting abilities observed for D5 were higher (*λ*_abs_ = 476 nm with *ε* = 45,900 M^− 1^ cm^− 1^ in ACN) than those for the furan counterpart (*λ*_abs_ = 439 nm with *ε* = 33,000 M^− 1^ cm^− 1^ in ACN). However, the performance of the solar cells based on the furan counterpart (ɳ_max_ = 7.36%) was better as compared to the one based on D5 (ɳ_max_ = 6.09%) because of the faster recombination lifetimes in D5. Again, the tendency of trapping of charge from the TPA moeity was higher in thiophene than the furan. In 2016, Simon et al. reported an enhancement in the photovoltage for DSSCs that employed triarylamine-based dyes, where halogen-bonding interactions existed between a nucleophilic electrolyte species (I^−^) and a photo-oxidized dye immobilized on a TiO_2_ surface. They found larger rate constants for dye regeneration (*k*_reg_) by the nucleophilic electrolyte species when heavier halogen substituents were positioned on the dye. Through the observations, they concluded that the halogen-bonding interactions between the dye and the electrolyte can boost the performance of DSSC [245]. However, the most efficient metal-free organic dye-based DSSC has shown PCE of 10.3% in combination with a cobalt redox shuttle, by using the phenyl dihexyloxy-substituted triphenylamine (TPA) (DHO-TPA) Y123 dye [246]. In 2018, Manfredi and group have designed di-branched dyes based on a triphenylamino (TPA) donor core with different aromatic and heteroaromatic peripheral groups bonded to TPA as auxiliary donors [247]. Thus, due to the improved strategic interface interactions between the dye sensitized titania and the liquid electrolyte, better optical properties were achieved.

##### Phenothiazine (PTZ) Dyes

Phenothiazine is a heterocyclic compound containing electron-rich sulfur and nitrogen heteroatoms, with a non-planar and butterfly conformation in the ground state, which can obstruct the molecular aggregation and the intermolecular excimer formation. Thus, PTZ results as a promising hole transport semiconductor in the organic devices, presenting unique electronic and optical properties [248].

Tian and co-workers investigated the effect of PTZ as an electron-donating unit in DSSCs, and because of the stronger electron donating tendency of PTZ unit than the TPA unit (0.848 and 1.04 V vs. the normal hydrogen electrode (NHE), respectively) [249], they found efficient results for the sensitizers based on PTZ rather than those based on the TPA [250]. In 2007, a new series of PTZ-based dyes as T2–1 to T2–4 was demonstrated [251]. In these dyes, PTZ unit acted as an electron donor, cyanoacrylic acid or rhodanine-3-acetic acid was used as an electron acceptor, and alkyl chains were used to increase the solubility. They found a red shift in the absorption spectra of T2–3 (*η* = 1.9%) and T2–4 (*η* = 2.4%) dyes with low IPCE values for rhodanine-3-acetic acid as an anchoring group, as compared to T2–1 (*η* = 5.5%) and T2–2 (*η* = 4.8%) dyes with cyanoacrylic acid as an anchoring group. This proved the use of the cyanoacrylic acid is more viable than a rhodanine-3-acetic acid. In 2010, Tian et al. reported modified phenothiazine (P1-P3) dyes [252] with the molecular structure containing the same acceptor and conjugation chain but different donors. Due to the presence of two methoxy groups attached to TPA, a red shift was observed in the absorption spectra of P1 as compared to P2 and P3. This resulted in an increment in the extent of electron delocalization over the whole molecule and, thus, a little red shift in the maximum absorption peak was observed. Xie et al. synthesized two novel organic dyes (PTZ-1 and PTZ-2) using electron-rich phenothiazine as electron donors and oligothiophene vinylene as conjugation spacers. They employed 13 μm transparent and 1.5 μm scattering TiO_2_ electrode and used an electrolyte composed of 0.6 M butylmethylimidazolium iodide (BMII), 0.03 M I_2_, 0.1 M GuSCN, 0.5 M 4-tert-butylpyridine in acetonitrile (TBP in ACN), and valeronitrile. They demonstrated that the (2E)-2-cyano-3-(5-(5-((E)-2-(10-(2-ethylhexyl)-10H-phenothiazin-7-yl)vinyl)thiophen-2-yl)thiophen-2-yl)acrylic acid (PTZ-1) and (2E)-3-(5-(5-(4,5-bis((E)-2-(10-(2-ethylhexyl)-10Hphenothiazin-3-yl)vinyl)thiophen-2-yl)thiophen-2-yl)thiophen-2-yl)-2cyanoacrylic acid (PTZ-2)-based DSSC showed *V*_OC_ = 0.70 V, *J*_SC_ = 11.69 mAcm^− 2^, FF = 65.3, and *η* = 5.4% and *V*_OC_ = 0.706 V, *J*_SC_ = 7.14 mAcm^− 2^, FF = 55.6, and *η* = 2.80% [150] under AM 1.5100 mWcm^−2^ illumination, respectively. The effect of hydrophilic sensitizer PTZ-TEG together with an aqueous choline chloride-based deep eutectic solvent (used as an electrolyte) has been reported [253]. In the study, glucuronic acid (GA) was used as a co-absorbent because it as has a simple structure and polar nature and is also able to better interact with hydrophilic media and components and possibly participates to the hydrogen bind interaction operated in the DES medium. PCE of 0.50% was achieved for the 1:1 dye/coabsorbent ratio.

##### Carbazole Dyes

It is a non-planar compound and can improve the hole transporting ability of the materials as well as avert the dye aggregate formation [235]. Due to its unique optical, electrical, and chemical properties, this compound has been applied as an active component in solar cells [254, 255]. Even with the addition of carbazole unit into the structure, the thermal stability and glassy state durability of the organic molecules were observed to be improved significantly [256, 257]. Tian et al. reported an efficiency of 6.02% for the DSSCs using S4 dye as a sensitizer, with an additional carbazole moiety to the outside of the donor group and found that the additional moiety facilitated the charge separation thereby decreasing the recombination rate between conduction band electrons and the oxidized sensitizer [185].

A series of MK-1, MK-2, and MK-3 dyes based on carbazole were reported by Koumura et al., where MK-1 and MK-2 have alkyl groups but MK-3 had no alkyl group. They showed that the presence of alkyl groups increased the electron lifetime and consequently *V*_OC_ in MK-1 and MK-2 [203, 258, 259], and due to the absence of alkyl groups, lower electron lifetime values could be responsible for the recombination process between the conduction band electrons and dye cations in MK-3. New structured dyes, i.e., D–A–π–A-type and D–D–π–A-type organic dyes, have been developed by inserting the subordinate donor–acceptor such as 3,6-ditert-butylcarbazole-2,3-diphenylquinoxaline to facilitate electron migration, restrain dye aggregation, and improve photostability [260]. Thus, by further extending the π conjugation of the linkers, mounting the electron-donating and electron-accepting capability of donors and acceptors, and substituting long alkyl chains, more stable DSSCs with lower dye aggregation and higher efficiency can be achieved.

##### Phenoxazine (POZ) Dyes

Phenoxazine is a tricyclic isoster of PTZ. The PTZ and POZ units display a stronger electron donating ability than the TPA unit (0.848, 0.880, and 1.04 V vs. normal hydrogen electrode (NHE), respectively) [261]. However, DSSCs based on POZ dyes show better cell performance as compared to PTZ dye-based DSSCs [261]. In 2009, two POZ-based dyes were demonstrated by Tian et al., i.e., a simple POZ dye TH301 and triphenylamine attached to TH301, named as TH305. Due to the insertion of TPA unit in TH305, a red shift in the absorption band was seen because of the higher electron donating capability of POZ. The efficiencies obtained for TH301 and TH305 were 6.2% and 7.7%, respectively, where standard N719 sensitizer showed an efficiency of 8.0% under similar conditions [206]. Thus, in 2011, Karslson reported a series of dyes MP03, MK05, MK08, MK12, and MK13, based on POZ unit, to increase the absorption properties of the sensitizers [261]. Further, two novel metal-free dyes (DPP-I and DPP-II) with a diketopyrrolopyrrole (DPP) core were synthesized for dye-sensitized solar cells (DSSCs) by Qu et al. [262]. They demonstrated the better photovoltaic performance with a maximum monochromatic IPCE of 80% and *η* = 4.14% with *J*_SC_ = 9.78 mAcm^− 2^, *V*_OC_ = 605 mV, and FF = 0.69, for the DSSC based on dye DPP-I.

Singh et al. have demonstrated nanocrystalline TiO_2_ dye-sensitized solar cells with PCE of 4.47% successfully designed two metal-free dyes (TPA–CN1–R2 and TPA–CN2–R1), containing triphenylamine and cyanovinylene 4-nitrophenyls as donors and carboxylic acid as an acceptor [263].

Semiconductor quantum dots (QDs) are another attractive approach to being sensitizers. These are II–VI and III–V type semiconductor particles whose size is small enough to produce quantum confinement effects. QD is a fluorescent semiconductor nanocrystal or nanoparticle typically between 10 and 100 atoms in diameter and confines the motion of electrons in conduction band, holes in valence band, or simply excitons in all three spatial directions. Thus, by changing the size of the particle, the absorption spectrum of such QDs can be easily varied. An efficiency of 7.0% has been recorded by collaborating groups from the University of Toronto and EPFL [264]. This recorded efficiency was higher than the solid-state DSSCs and lower than the DSSCs based on liquid electrolytes. A high performance QDSSC with 4.2% of PCE was demonstrated by Li et al. This cell consisted of TiO_2_/CuInS_2_-QDs/CdS/ZnS photoanode, a polysulfide electrolyte, and a CuS counter electrode [265]. In 2014, a conversion efficiency of 8.55% has been reported by Chuang et al. [266]. Recently, Saad and co-workers investigated the influence on the absorbance peak on N719 dye due to the combination between cadmium selenide (CdSe) QDs and zinc sulfide (ZnS) QDs [267]. The cyclic voltammetry (CV) of varying wt% of ZnS found that the 40 wt% of ZnS is an apposite combination for a DSSC’s photoanode and has produced the higher current. However, 50 wt% of ZnS was found to be the best concerto to increase the absorbance peak of the photoanode.

#### Natural dyes

New dye materials are also under extensive research, due to the intrinsic properties of Ru(II)-based dyes, and as a result to replace these rare and expensive Ru(II) complexes, the cheaper and environmentally friendly natural dyes overcome as an alternative [268].

Natural dyes provide low-cost and environmentally friendly DSSCs. There are various natural dyes containing anthocyanin [268], chlorophyll [269], flavonoid [270], carotenoid [271], etc. which have been used as sensitizers in DSSCs. Table 5 provides the general characteristics of these dyes, i.e., their availability and color range.

**Table 5:** Availability and color range for the natural dyes (anthocyanin, carotenoid, chlorophyll, and flavonoid)

Sensitizer	Availability	Color range	Reference
Anthocyanin	Flowers, fruits, leaves, roots, tubers, and stems of the plant	Purple red	^(a)^ [403] ^(b)^ [404]
Carotenoid	Fruits, flowers of plants, and microorganisms	(a) Red, yellow, and orange colors to flowers and fruits (b) Yellow to orange petal colors	^(a)^ [405] ^(b)^ [271]
Chlorophyll	Leaves of mostly green plants, algae, and cyanobacteria	Green	[275]
Flavonoid	Plants including angiosperms, gymnosperms, ferns, and bryophytes	Various colors of flavonoids are determined by the degree of oxidation of the C-ring	[273]

##### Molecular Structure

**Fig. 23:**
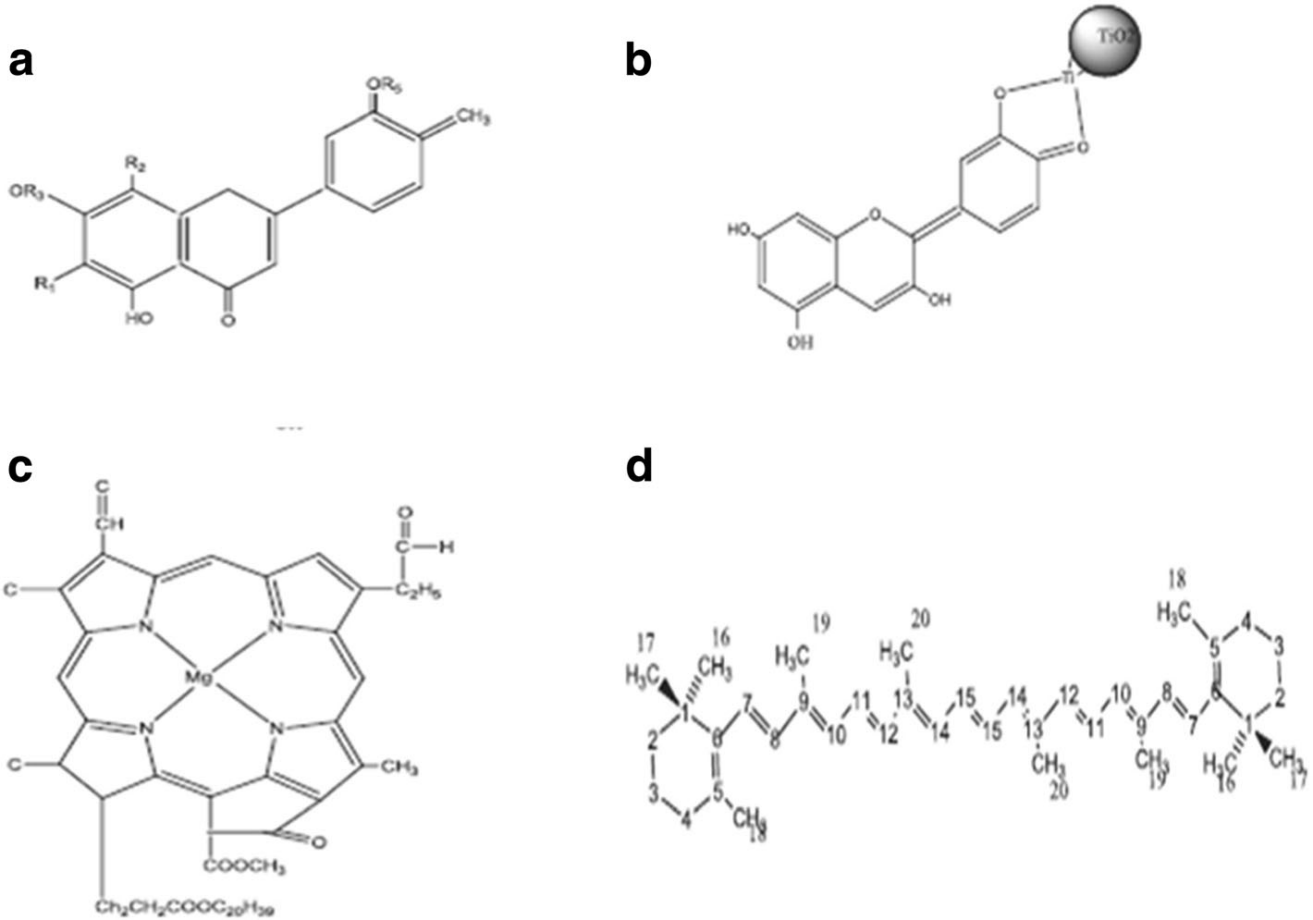
Chemical structures of **a** anthocyanin, **b** flavonoid, **c** β,β-carotene, and **d** chlorophyll

*Anthocyanin*: The molecular structure of anthocyanin is shown in Fig. 23a. In anthocyanin molecule, the carbonyl and hydroxyl groups are bound to the semiconductor (TiO_2_) surface, which stimulates the electron transfer from the sensitizer (anthocyanin molecules) to the conduction band of porous semiconducting (TiO_2_) film. Anthocyanin can absorb light and transfer that light energy by resonance energy transfers to the anthocyanin pair in the reaction center of the photosystems [272].

*Flavonoid*: Flavonoid is an enormous compilation of natural dyes which shows a carbon framework (C_6_–C_3_–C_6_) or more particularly the phenylbenzopyran functionality, as shown in Fig. 23b [273]. It contains 15 carbons with two phenyl rings connected by three carbon bridges, forming a third ring, where the different colors of flavonoids depend on the degree of phenyl ring oxidation (C-ring). Its adsorption onto mesoporous TiO_2_ surface is quite fast by displacing an OH counter ion from the Ti sites that combines with a proton donated by the flavonoid [274].

*Carotenoid*: Andanthocyanin, flavonoids, and carotenoids are often found in the same organs [275]. Carotenoids are the compounds having eight isoprenoid units that are widespread in nature (as shown in Fig. 23c). Beta-carotene dye has an absorbance in wavelength zones from 415 to 508 nm, has the largest photoconductivity of 8.2 × 10^− 4^ and 28.3 × 10^− 4^ (Ω.m)^− 1^ in dark and bright conditions [276], and has great potential as energy harvesters and sensitizers for DSSCs [277].

*Cholorophyll:* Among six different types of chlorophyll pigments that actually exist, Chl α is the most occurring type. Its molecular structure comprises a chlorine ring with a Mg center, along with different side chains and a hydrocarbon trail, depending on the Chl type (as shown in Fig. 23d).

In 1997, antocyanins extracted from blackberries gave a conversion efficiency of 0.56% [268]. The roselle (*Hibiscus sabdariffa* containing anthocyanin) flowers and papaw (*Carica papaya* containing chlorophyll) leaves were also investigated as natural sensitizers for DSSCs. Eli et al. sensitized TiO_2_ photoelectrode with roselle extract (*η* = 0.046%) and papaw leaves (*η* = 0.022%), respectively and found better efficiency for roselle extract-sensitized cell because of the broader absorption of the roselle extract onto TiO_2_ [278]. Tannins have also been attracted as a sensitizer in DSSCs due to their photochemical stability. DSSCs using natural dyes tannins and other polyphenols (extracted from Ceylon black tea) have given photocurrents of up to 8 mAcm^− 2^ [168]. Haryanto et al. fabricated a DSSC using annato seeds (*Bixa orellana Linn*) as a sensitizer [279]. They demonstrated *V*_OC_ and *J*_SC_ for 30 g, 40 g, and 50 g as 0.4000 V, 0.4251 V, and 0.4502 V and 0.000074 A, 0.000458 A, and 0.000857 A, respectively. The efficiencies of the fabricated solar cells using annato seeds as a sensitizer for each varying mass were 0.00799%, 0.01237%, and 0.05696%. They observed 328–515 nm wavelength range for annato seeds with the help of a UV-vis spectrometer. Hemalatha et al. reported a PCE of 0.22% for the *Kerria japonica* carotenoid dye-sensitized solar cells in 2012 [280].

In 2017, a paper was published on DSSCs sensitized with four natural dyes (viz. Indian jamun, plum, black currant, and berries). The cell achieved highest PCE of 0.55% and 0.53%, respectively, for anthocyanin extracts of blackcurrant and mixed berry juice [281]. Flavonoid dye extracted from Botuje (*Jathopha curcas Linn*) has been used a sensitizer in DSSCs. Boyo et al. achieved *η* = 0.12% with the *J*_SC_ = 0.69 mAcm^− 2^, *V*_OC_ = 0.054 V, and FF = 0.87 for the flavonoid dye-sensitized solar cell [282]. Bougainvillea and bottlebrush flower can also be used as a sensitizer in DSSCs because both of them show a good absorption level in the range of 400 to 600 nm as a sensitizer, with peak absorption at 520 nm for bougainvillea and 510 nm for bottlebrush flower [283]. A study of color stability of anthocyanin (mangosteen pericarp) with co-pigmentation method has been conducted by Munawaroh et al. They have found higher color retention for anthocyanin-malic acid and anthocyanin-ascorbic acid than that of pure anthocyanin [284]. Thus, the addition of ascorbic acid and malic acid as a co-pigment can be performed to protect the color retention of anthocyanin (mangosteen pericarp) from the degradation process. The *I*–*V* characteristics of DSSCs employing different natural dyes are shown in Table 6.

**Table 6:** PV characteristics for different natural dye-sensitized solar cells

Dye	Result	*J*_SC_ (mAcm^−2^)	*V*_OC_ (*V*)	FF	*η* (%)	Reference
Roselle	Absorption peak of the photoanode was broader than that of the dye solution due to the binding of anthocyanin in the extract to the TiO_2_ surface with a shift to a higher wavelength (from 540 to 560 nm)	0.18	0.47	0.55	0.046	[278]
Red Cabbage	Absorption band and intensity has observed to be enhanced due to the interfacial Ti–O coupling between the dye molecule and the TiO_2_ molecules	4.38	0.47	0.36	0.73	[406]
Morinda lucida	Shows absorption maxima at 600 nm and 440 nm	2.56	0.44	0.47	0.53	[407]
Sumac/Rhus	Visible absorption band shifts to higher energy, showing a maximum absorption around 400–500 nm upon adsorption onto TiO_2_	0.93	0.39	0.41	1.5	[408]
*Hibiscus rosa-sinensis*	–	0.96	0.26	0.43	0.11	[409]
Mangosteen peel	Absorption spectrum of mangosteen peel dye on TiO_2_ showed absorption at wavelengths ranging from 350 to 550 nm	8.70	0.60	0.50	2.63	[104]
Papaya leaves	Molar extinction coefficient was found to be 86,300 M^−1^ cm^−1^ at 660 nm	0.402	0.56	0.41	0.094	[410]
Dragon fruit	Absorption spectrum showed peak value of 535 nm and found intermolecular H-bond, conjugate C=O stretching and esters acetates C–O–C stretching vibration, due to the component of anthocyanin	0.20	0.22	0.30	0.22	[411]
Red rose	Maximum absorption for red rose was found at 535 nm and maximum absorption coefficient was about 15 times higher than that of the N719 dye	4.57	0.48	0.36	0.81	[412]
*Lawsonia inermis* leaves	Showed absorption maxima at 518 nm due to higher solubility in ethanol	1.87	0.61	0.58	0.66	[413]
*E. conferta*	Showed a broad maximum around 530–560 nm with maximum absorption at 540 nm	4.63	0.37	0.56	1.00	[414]
*G. atroviridis*	Absorption peaks were observed to be between 540 and 550 nm with maximum absorption at 540 nm	2.55	0.32	0.63	0.51	[414]
Sweet pomegranate	Maximum absorption of the dye onto TiO_2_ was found at 536 nm	4.60	0.62	0.55	1.57	[415]
Cosmos	Peaks were observed at about 505 and 590 nm of wavelengths, respectively verifying the charge injection from the excited state of the natural sensitizer	1.041	0.447	0.61	0.54	[274]
Golden trumpet	Positive shift in the absorption peak was observed after adsorption	0.878	0.405	0.54	0.40	[274]
JDND2	Jackfruit derived natural dye (JDND) exhibited overriding photo-absorption in a spectral range of 350–800 nm with an optical bandgap of ∼ 1.1 eV	2.21	0.805	0.60	1.07	[416]
Indian jamun	An improvement in ideality factor (A) was observed 4.8 for Jamun dye-based DSSC	1.56	0.580	0.58	1.23	[417]
*Nephelium lappaceum*	–	3.88	0.404	0.35	0.56	[418]
Tamarillo fruit	Tamarillo pulp showed highest absorbance in the visible light wavelength of ~ 450–560 nm	0.356	0.542	–	0.043	[419]
Chlorophyll	–	0.145	0.585	0.59	0.055	[310]
Xanthophyll	Xanthophyll dye showed more stable (shows low degradation over a 24-h period) than chlorophyll under light, but concentration of the adsorbed xanthophyll pigments was found to be 2.5 × 10^−4^ μg/ml, much lesser than that of chlorophyll pigments (2.2 μg/ml)	0.104	0.610	0.54	0.038	[310]
Kenaf Hibiscus	UV spectra showed a peak at 378 nm and a small hump at 554 nm, whereas dye absorbed TiO_2_ film showed two peaks at 385 nm and 548 nm with broad absorption spectra in the visible range as required for a solar cell	6.6733	0.478	0.60	2.87	[420]

#### Organic Complexes of Other Metals

  Os, Fe and Pt complexes [285, 286, 287] are considered to be some other promising materials in DSSCs. Besides the fact that Os complexes are highly toxic, they are applied as a sensitizer in DSSCs due to its intense absorption (α811nm = 1.5 × 103 M− 1 cm− 1) and for the utilization of spin forbidden singlet-triplet MLCT transition in the NIR. Higher IPCE values were obtained in this spectral region; however, the overall conversion efficiency was only 50% of a standard Ru dye. Pt complexes have given modest efficiencies of ca. 0.64% [286] a and iron complexes, which are very interesting due to the vast abundance of the metal and its non-toxicity; the solvatochromism of complexes like [FeIIL2(CN)2] can be used to adjust their ground and excited state potentials and increase the driving force for electron injection into the semiconductor conduction band or for regeneration of the oxidized dye by the electrolyte couple [287].

Thus, a number of metal dyes, metal-free organic dyes, and natural dyes have been synthesized till today. Many other dyes like K51 [288], K60 [289], K68 [290]; D5, D6 (containing oligophenylenevinylene π-conjugated backbones, each with one *N*,*N*-dibutylamino moiety) [291]; K77 [292]; SJW-E1 [293]; S8 [294]; JK91 and JK92 [295]; CBTR, CfBTR, CiPoR, CifPoR, and CifPR [296, 297]; Complexes A1, A2, and A3 [298]; T18 [299]; A597 [300]; YS-1–YS-5 [301]; YE05 [302]; and TFRS-1–3 [303] were developed and applied as sensitizers in DSSCs.

## Latest Approaches and Trends

**Fig. 24:**
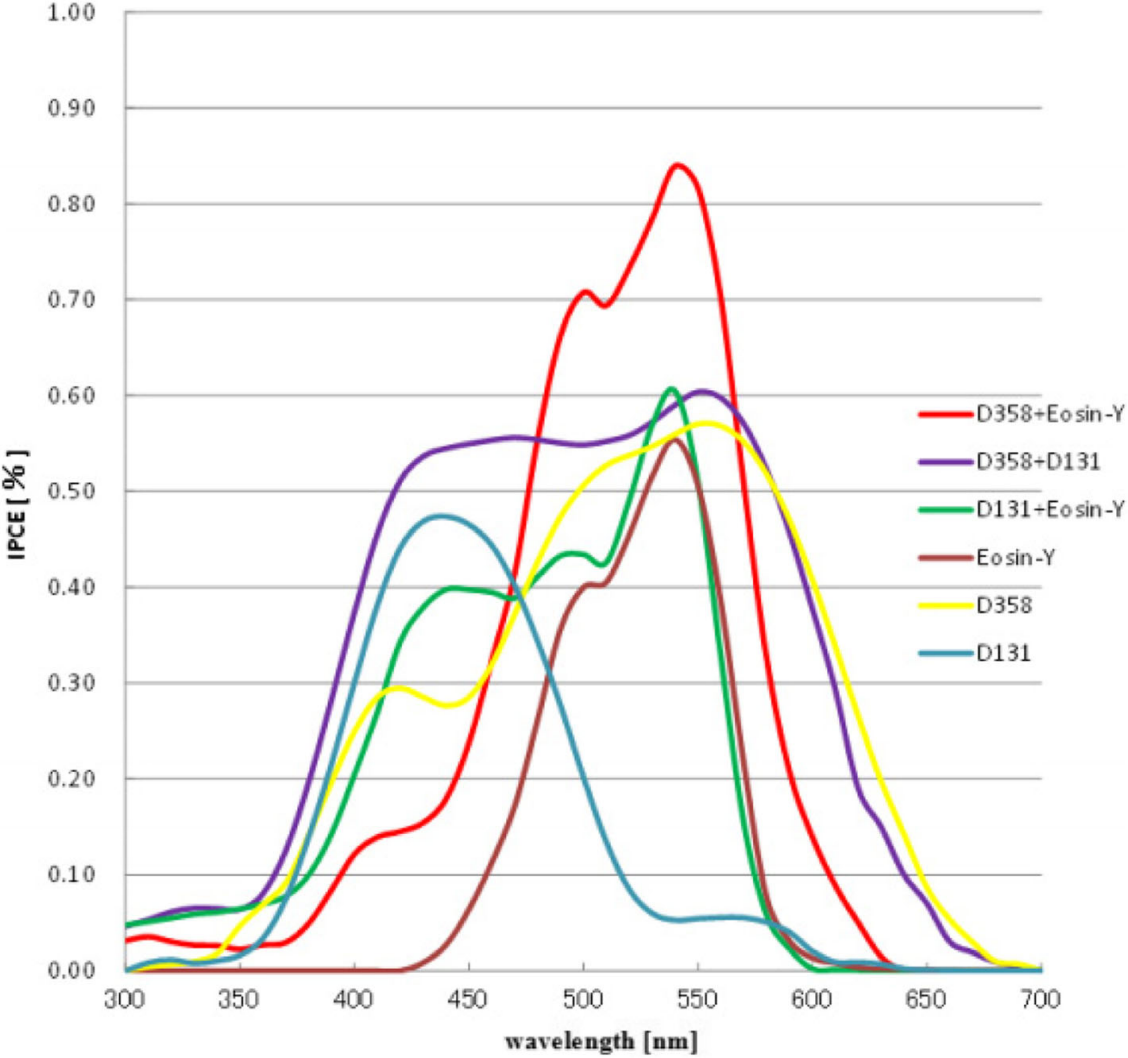
IPCE of mixed pigment and single pigments, where single pigment were Eosin Y, D131, and D358 and mixed pigments were D358 and Eosin Y; D358 and D131; D131 and Eosin Y [311]

However, a different trend to optimize the performance of the DSSCs has been started by adding the energy relay dyes (ERDs) to the electrolyte [57, 304]; inserting phosphorescence or luminescent chromophores, such as applying rare-earth doped oxides [58–60] into the DSSC; and coating a luminescent layer on the glass of the photoanode [61, 62]. In the process of adding the ERDs to the electrolyte or to the HTM, some highly luminescent fluorophores have to be chosen. The main role of ERD molecules in DSSCs is to absorb the light that is not in the primary absorption spectrum range of the sensitizing dye and then transfer the energy non-radiatively to the sensitizing dyes by the fluorescence (Forster) resonance energy transfer (FRET) effect [305]. An improvement in the external quantum efficiency of 5 to 10% in the spectrum range from 400 to 500 nm has been demonstrated by Siegers and colleagues [306]. Recently, Lin et al. reported the doping of 1,8-naphthalimide (N-Bu) derivative fluorophore directly into a TiO_2_ mesoporous film with N719 for application in DSSCs [307], in which the N-Bu functioned as the FRET donor and transferred the energy via spectral down-conversion to the N719 molecules (FRET acceptor). An improvement of the PCE from 7.63 to 8.13% under 1 sun (AM 1.5) illumination was attained by the cell. Similarly, Prathiwi et al. fabricated a DSSC by adding a synthetic dye into the natural dye containing anthocyanin (from red cabbage) in 2017 [308]. They prepared two different dyes at different volumes, i.e., anthocyanin dye at a volume of 10 ml and combination dyes at a volume of 8 ml (anthocyanin): 2 ml (N719 synthetic dye), respectively. They observed an enhancement in conversion efficiency up to 125%, because individually the anthocyanin dye achieved a conversion efficiency of 0.024% whereas for the combination dye 0.054% conversion efficiency was achieved. This enhancement was considered due to the higher light absorption. Thus, greater photon absorption took place and the electrons in excited state were also increased to enhance the photocurrent. Thus, cocktail dyes are also developing as a new trend in DSSCs. Chang et al. achieved a *η* = 1.47% when chlorophyll dye (from wormwood) and anthocyanin dye (from purple cabbage) as natural dyes were mixed together at volume ratio of 1:1 [309], whereas the individual dyes showed lower conversion efficiencies. Puspitasari et al. fabricated different DSSCs by mixing the three different natural dyes as turmeric, mangosteen, and chlorophyll. The highest efficiency of 0.0566% was attained for the mixture of the three dyes, where the absorbance peak of the mixed dyes was observed at 300 nm and 432 nm [106]. Similarly, Lim and co-workers have achieved a 0.085% of efficiency when mixing the chlorophyll and xanthophyll dyes together [310]. In 2018, Konno et al. studied the PV characteristics of DSSCs by mixing different dyes and observed highest *ɳ* = 3.03% for the combination dye “D358 + D131,” respectively [311]. Figure 24 shows the IPCE of mixed pigments and single pigments.

**Fig. 25:**
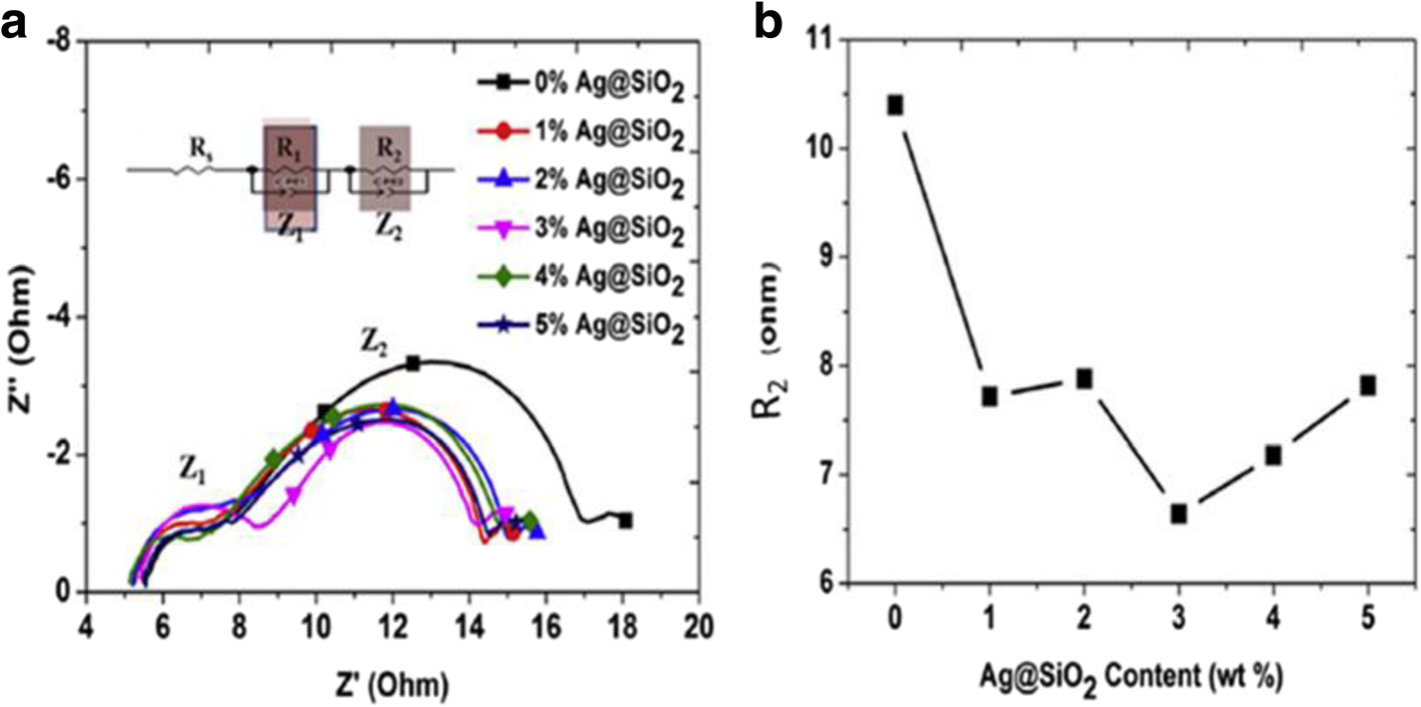
**a** Nyquist plots obtained from the EIS of DSSCs with varying Ag@SiO_2_ content (inset shows the equivalent circuit). **b** R2 ohm with respect to the Ag@SiO_2_ NPs content [313]

**Fig. 26:**
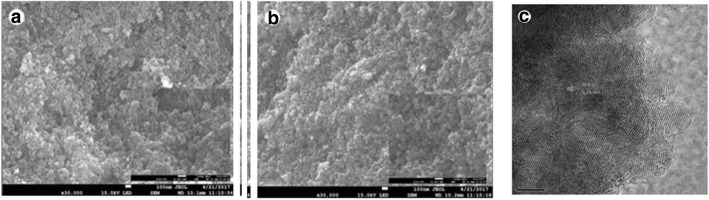
SEM images of **a** CdS/CdSe and **b** CdS/Mn:CdSe QD sensitization on TiO^2^ surface. **c** TEM image of CdS/Mn:CdSe QDs [318]

An approach used to enhance the performance of DSSCs is plasmonic effect. Surface plasmon resonance (SPR) is resonant oscillation of conduction electrons at the interface between negative and positive permittivity material stimulated by incident light. In 2013, Gangishetty and co-workers synthesized core-shell NPs comprising a triangular nanoprism core and a silica shell of variable thickness. SPR band centered at ~ 730 nm was observed for the nanoprism Ag particles, which overlapped with the edge of the N719 absorption spectrum very well. They found the incorporation of the nanoprism Ag particles into the photoanode of the DSSCs yielded a 32% increase in the overall PCE [312]. Hossain et al. used the phenomenon of plasmonic with different amounts of silver nanoparticles (Ag NPs) coated with a SiO_2_ layer prepared as core shell Ag@SiO_2_ nanoparticles (Ag@SiO_2_ NPs) and studied the effect of SiO_2_-encapsulated Ag nanoparticles in DSSCs. They found the highest PCE of 6.16% for the photoanode incorporated 3 wt% Ag@SiO_2_; the optimal PCE was 43.25% higher than that of a 0 wt% Ag@SiO_2_ NP photoanode [313]. However, a simultaneous decrease in the efficiency with further increases in the wt% ratio, i.e., for 4 wt% Ag@SiO_2_ and 5 wt% Ag@SiO_2_, was observed. This decrease for the excess amounts of Ag@SiO_2_ NPs was attributed to three reasons: (i) reduction in the effective surface area of the films, (ii) absorption of less amount of the dye, and (iii) an increase in the charge-carrier recombination [314]. After analyzing the nyquist plots (as shown in Fig. 25), they have found a decreased diameter of Z_2_ monotonically as the Ag@SiO_2_ NP content increased to 3 wt% and R2 decreased from 10.4 to 6.64 Ω for the conventional DSSC to the 3 wt% Ag@SiO_2_ NPs containing DSSC. Jun et al. used quantum-sized gold NPs to create plasmonic effects in DSSCs [315]. They fabricated the TiO_2_ photoanode by incorporating the Au nanoparticles (Au NPs) with an average diameter of 5 nm into the commercial TiO_2_ powder (average diameter 25 nm) and used N749 black dye as a sensitizer. Thus, due to the SPR effect, the efficiency for the DSSC (incorporating Au NPs) was enhanced by about 50% compared to that without Au nanoparticles. Effect of incorporating green-synthesized Ag NPs into the TiO_2_ photoanode has been investigated in 2017 [316]. Uniform Ag NPs synthesized by treating silver ions with *Peltophorum pterocarpum* flower extract at room temperature showed the Ag NPs as polycrystalline in nature with face centered cubic lattice with an approximate size in the range of 20–50 nm [316]. The PCE of the device was improved from 2.83 to 3.62% with increment around 28% after incorporation of the 2 wt% of the Ag NPs due to the plasmonic effect of the modified electrode. Bakr et al. have fabricated Z907 dye-sensitized solar cell using gold nanoparticles prepared by pulsed Nd:YAG laser ablation in ethanol at wavelength of 1064 nm [63]. The addition of synthesized Au NPs to the Z907 dye increased the absorption of the Z907 dye, thus achieving *ɳ* = 1.284% for the cell without Au NPs and 2.357% for the cell incorporating the Au NPs. Recently, in 2018, a novel 3-D transparent photoanode and scattering center design was applied as to increase the energy conversion efficiency from 6.3 to 7.2% of the device [317] because the plasmonics plays an important role in the absorption of light and thus, the application is developing at a very fast pace and grabbing a lot of attention worldwide in the last few years. Recently, a study on incorporation of Mn^2+^ into CdSe quantum dots was carried out by Zhang and group [318]. An improved efficiency from 3.4% (CdS/CdSe) to 4.9% (CdS/Mn-CdSe) was achieved for the device upon the addition of Mn^2+^ into CdSe because when Mn^2+^ is doped into the CdSe (as shown in Fig. 26), the QDs on the surface of the film became compact and the voids among the particles were small, thus reducing the recombination of photogenerated electrons. Also with the loading of Mn^2+^ into the CdSe, the size of the QD clusters was increased. However, in QDSCs (quantum dot-sensitized solar cells), there is an inefficient transfer of electrons through the mesoporous semi-conductor layer [319], because their application on a commercial level is still far off. Thus, Surana et al. reported the assembling of CdSe QDs, tuned for photon trapping at different wavelengths in order to achieve an optimum band alignment for better charge transfer in QDSC [319]. TiO_2_ hollow spheres (THSs) synthesized by the sacrifice template method was reported as a scattering layer for a bi-layered photoanode for DSSCs by Zhang and co-workers [320]. They used the mixture of multi-walled carbon nanotubes with P25 as an under layer and THSs as an overlayer for the photoanode which showed good light scattering ability. The cross-sectional FESEM images revealed the disordered mecroporous network for the scattering layer containing THSs which was supposed to be responsible for the enhanced light absorption and the transfer of electrolyte. Thus, *ɳ* = 5.13% was achieved for P25/MWNTs-THSs, whereas 4.49% of efficiency was reported for a pure P25 photoanode-based DSSC. Also, the electron lifetime (*τ*_e_) estimated for pure P25 by Bode phase plots of EIS spectra was 5.49 ms; however, 7.96 ms was shown for P25/MWNTs-THSs.

John and group reported the synthesis and application of ZnO-doped TiO_2_ nanotube/ZnO nanoflake heterostructure as a photoanode in DSSCs for the first time in 2016 [321]. They used different characterization techniques to investigate the layered structure of the novel nanostructure. The Rutherford backscattering spectroscopy revealed that during the doping process, a small percentage of Zn was doped into TONT in addition to the formation of ZnO nanoflakes on the top, which led to a preferential orientation of the nanocrystallites in the tube on annealing. Back in 2017, Zhang et al. reported paper on low-dimensional halide perovskite and their applications in optoelectronics due to the ~ 100% of photoluminescence quantum yields of perovskite quantum dots [322]. The main emphasis of their paper was on the study of halide perovskites and their versatile application, i.e., in optoelectronics in spite of PV applications only. The main role of perovskite nanoparticles in solar cells is being applied as sensitizers. Similarly, in the queue of developing highly efficient DSSCs, Chiang and co-workers fabricated DSSCs based on PtCoFe nanowires with rich {111} facets exhibiting superior I^−^ _3_ reduction activity as a counter electrode, which surpassed the previous PCE record of the DSSCs using Ru(II)-based dyes [323]. Recently, in accordance with enhancing the charge collection efficiencies (*η*_coll_) as well as PCE of DSSCs, Kunzmann et al. reported a new strategy of fabricating low-temperature (lt)-sintered DSSC and demonstrated the highest efficacy reported for lt-DSSC to date [324]. They have integrated TiO_2_-Ru(II) complex (TiO_2__Ru_IS)-based hybrid NPs into the photoelectrode. Due to a better charge transport and a reduced electron recombination, devices with single-layer photoelectrodes featuring blends of P25 and TiO_2__Ru_IS give rise to a 60% *η*_coll_ relative to a 46% *η*_coll_ for devices with P25-based photoelectrodes. Further, for usage of a multilayered photoelectrode architecture with a top layer based on TiO_2__Ru_IS only, devices with an even higher *η*_coll_ (74%) featuring a *η* = 8.75% and stabilities of 600 h were shown. The two major rewards obtained for such devices were the dye stability due to its amalgamation into the TiO_2_ anatase network and, secondly, the enhanced charge collection yield due to its significant resistance towards electron recombination with electrolytes.

## Conclusions

The main aim of this study was to put a comprehensive review on new materials for photoanodes, counter electrodes, electrolytes, and sensitizers as to provide low-cost, flexible, environmentally sustainable, and easy to synthesize DSSCs. However, a brief explanation has been given to greater understand the working and components of DSSCs. One of the important emphases in this article has been made to establish a relation between the photosensitizer structure, the interfacial charge transfer reactions, and the device performance which are essential to know as to develop new metal and metal-free organic dyes. In terms of low stability offered by DSSCs, two major issues, i.e., low intrinsic stability and the sealing of the electrolytes (extrinsic stability), have been undertaken in this study. To fulfill huge demand of electricity and power, we have two best possible solutions: this demand should be compensated either by the nuclear fission or by the sun. Even so, the nuclear fission predicted to be the best alternative has great environmental issues as well as a problems associated with its waste disposal. Thus, the second alternative is better to follow. DSSCs are developed as a cheap alternative but the efficiency offered by DSSCs in the field is not sufficient. Thus, we have to do a wide research on all possible aspects of DSSCs. We proposed to develop DSCCs based on different electrodes viz. graphene, nanowires, nanotubes, and quantum dots; new photosensitizers based on metal complexes of Ru or Os/organic metal-free complexes/natural dyes; and new electrolytes based on imidazolium salts/pyridinium salts/conjugated polymers, gel electrolytes, polymer electrolytes, and water-based electrolytes. In summary, so far, extensive studies have been carried out addressing individual challenges associated with working electrode, dye, and electrolytes separately; hence, a comprehensive approach needs to be used where all these issues should be addressed together by choosing appropriate conditions of electrolyte (both in choice of material and structure), optimum dye, and the most stable electrolyte which provides better electron transportation capability.

In terms of their commercial application, a DSSC needs to be sustainable for > 25 years in building-integrated modules to avoid commotion of the building environment for repair or replacement and a lifespan of 5 years is sufficient for portable electronic chargers integrated into apparel and accessories [325]. However, DSSCs are being quite bulky due to their sandwiched glass structure, but the flexible DSSCs (discussed elsewhere) that can be processed using roll-to-roll methods may came as an alternative but then has to compromise with the shorter lifespan. Although the stability and lifetime of a DSSC most probably depend on the encapsulation and sealing as discussed above. Apart from the usage of expensive glass substrates in the case of modules and panels, one of the biggest hurdles is to manufacture glass that is flat at the 10 μm length scale over areas much larger than 30 × 30 cm^2^ [326] and the humidity. Another challenge is to choose which metal interconnects in the cells that are more or less corroded to the electrolyte, and high degree of control over cell-to-cell reproducibility is required to achieve same current and/or voltage for all the cells in the module. If the abovementioned challenges would be overcome, then there is no roadblock for the commercial applications of DSSCs, which has been restricted up to an amicable extent. G24i has introduced a DSC module production of 25 MW capacity in 2007 in Cardiff, Wales (UK), with extension plans up to 200 MW by the end of 2008 (http://www. g24i. com), and afterwards, many DSSC demonstration modules are now available. However, the maximum outdoor aging test of DSSCs is reported for 2.5 years up to now [327].

## Abbreviations

ACN: Acetonitrile
Ag NPs: Silver nanoparticles
AM 1.5: Air mass 1.5
Au NPs: Gold nanoparticles
BODIPY: Boradiazaindacene
BPI: 4,5-Bis(4-methoxyphenyl)-1H-imidazole
C: Carbon
CBZ: Carbazole
CDCA: Chenodeoxycholic acid
CdSe: Cadmium selenide
CNF: Carbon nanofiber
CNT: Carbon nanotube
CPEs: Conjugated polymer electrolytes
CuBr: Copper bromide
CuI: Copper iodide
CuSCN: Copper thiocyanate
CV: Cyclic voltammetry
DPP: Diketopyrrolopyrrole
DSSCDB: Dye-sensitized solar cell database
DSSCs: Dye-sensitized solar cells
EC: Ethylene carbonate
EIS: Electron impedance spectroscopy
EPFL: Ecole Polytechnique Fédèrale de Lausanne
ERDs: Energy relay dyes
FF: Fill factor
FRET: Fluorescence (Forster) resonance energy transfer
FTO: Fluorine-doped tin oxide
GA: Glucuronic acid
GBL: γ-Butyrolactone
GuSCN: Guanidinium thiocyanate
HfO_2_: Hafnium oxide
HOMO: Highest occupied molecular orbital
HTMs: Hole transport materials
IL: Ionic liquid
IMVS: Intensity-modulated photovoltage spectroscopy
IPCE: Incident photon to current conversion efficiency
Ir: Iridium
ITO: Indium-doped tin oxide
*J*_SC_: Short circuit current
LCs: Liquid crystals
LHE: Light harvesting efficiency
LUMO: Lowest unoccupied molecular orbital
MePN: 3-Methoxypropionitrile
MLCT: Metal to ligand charge transfer
nc: Nanocrystalline
NHE: Normal hydrogen electrode
NIR: Near-infrared region
NMBI: *N*-Methylbenzimidazole
NMP: *N*-Methylpyrrolidine
NPs: Nanoparticles
Os: Osmium
PANI: Polyaniline
PC: Propylene carbonate
PCE: Power conversion efficiency
PEO: Poly(ethylene oxide)
PET: Polyethylene terephthalate
*P*_max_: Maximum power output
POZ: Phenoxazine
Pt: Platinum
PTZ: Phenothiazine
PV: Photovoltaic
QDs: Quantum dots
QSSE: Quasi-solid-state electrolyte
RT: Room temperature
Ru: Ruthenium
SCE: Saturated calomel electrode
SCs: Solar cells
SPR: Surface plasmon resonance
SSE: Solid-state electrolyte
TBP: 4-Tert-butylpyridine
TCO: Transparent conducting oxide
TiO_2_: Titanium dioxide
TPA: Triphenylamine
UV-vis: Ultraviolet-visible
*V*_OC_: Open circuit voltage
WE: Working electrode
ZnO: Zinc oxide
ZnS: Zinc sulfide
*η*: Efficiency
